# Extensive Natural Variation in Arabidopsis Seed Mucilage Structure

**DOI:** 10.3389/fpls.2016.00803

**Published:** 2016-06-07

**Authors:** Cătălin Voiniciuc, Eva Zimmermann, Maximilian Heinrich-Wilhelm Schmidt, Markus Günl, Lanbao Fu, Helen M. North, Björn Usadel

**Affiliations:** ^1^Institute for Bio- and Geosciences (IBG-2: Plant Sciences), Forschungszentrum JülichJülich, Germany; ^2^Institute for Botany and Molecular Genetics, BioEconomy Science Center, RWTH Aachen UniversityAachen, Germany; ^3^Centre National de la Recherche Scientifique, Institut Jean-Pierre Bourgin, INRA, AgroParisTech, Université Paris-SaclayVersailles, France

**Keywords:** Arabidopsis, plant cell wall, seeds, mucilage, pectin, hemicellulose, cellulose, galactoglucomannan

## Abstract

Hydrated *Arabidopsis thaliana* seeds are coated by a gelatinous layer called mucilage, which is mainly composed of cell wall polysaccharides. Since mucilage is rich in pectin, its architecture can be visualized with the ruthenium red (RR) dye. We screened the seeds of around 280 Arabidopsis natural accessions for variation in mucilage structure, and identified a large number of novel variants that differed from the Col-0 wild-type. Most of the accessions released smaller RR-stained capsules compared to the Col-0 reference. By biochemically characterizing the phenotypes of 25 of these accessions in greater detail, we discovered that distinct changes in polysaccharide structure resulted in gelatinous coatings with a deceptively similar appearance. Monosaccharide composition analysis of total mucilage extracts revealed a remarkable variation (from 50 to 200% of Col-0 levels) in the content of galactose and mannose, which are important subunits of heteromannan. In addition, most of the natural variants had altered Pontamine Fast Scarlet 4B staining of cellulose and significantly reduced birefringence of crystalline structures. This indicates that the production or organization of cellulose may be affected by the presence of different amounts of hemicellulose. Although, the accessions described in this study were primarily collected from Western Europe, they form five different phenotypic classes based on the combined results of our experiments. This suggests that polymorphisms at multiple loci are likely responsible for the observed mucilage structure. The transcription of *MUCILAGE-RELATED10* (*MUCI10*), which encodes a key enzyme for galactoglucomannan synthesis, was severely reduced in multiple variants that phenocopied the *muci10-1* insertion mutant. Although, we could not pinpoint any causal polymorphisms in this gene, constitutive expression of fluorescently-tagged MUCI10 proteins complemented the mucilage defects of a *muci10*-like accession. This leads us to hypothesize that some accessions might disrupt a transcriptional regulator of *MUCI10*. Therefore, this collection of publicly-available variants should provide insight into plant cell wall organization and facilitate the discovery of genes that regulate polysaccharide biosynthesis.

## Introduction

Due to their great abundance in nature, plant cell wall polysaccharides represent a potential resource for the sustainable production of biofuels and other valuable chemicals (Loqué et al., 2015). Despite this, major challenges must be addressed for cell wall conversion to become economically viable. Improved understanding of polysaccharide biosynthesis at the molecular level could provide new gene targets for the engineering of cell walls with improved properties for industrial applications.

The epidermal cells of the *Arabidopsis thaliana* seed coat represent a particularly attractive model for identifying genes involved in cell wall production (Haughn and Western, 2012). They accumulate copious amounts of hydrophilic polysaccharides, which are released upon hydration of mature seeds as a sticky capsule of mucilage (North et al., 2014). The structure of mucilage can be conveniently visualized with light microscopy, and mucilage can be easily extracted for biochemical analyses of cell wall composition. Pectin (primarily unbranched rhamnogalacturonan I; RG I) encapsulates hydrated seeds and is readily stained with ruthenium red (RR; Hanke and Northcote, 1975). Only 35% of the total RG I produced is part of the adherent mucilage layer that remains attached to seeds after gentle shaking in water (Voiniciuc et al., 2015c).

Adherent mucilage is at least partially anchored by cellulosic rays (Harpaz-Saad et al., 2011; Mendu et al., 2011; Sullivan et al., 2011; Griffiths et al., 2014), which can be stained with Pontamine Fast Scarlet 4B (S4B; Anderson et al., 2010), and are found to be birefringent under polarized light (Sullivan et al., 2011; Ben-Tov et al., 2015). Arabidopsis seed coat epidermal cells also produce other polysaccharides that play important roles despite their low abundance (Voiniciuc et al., 2015c). Galactoglucomannan (GGM), elongated by CELLULOSE SYNTHASE-LIKE A2 (CSLA2; Yu et al., 2014) and decorated by MUCILAGE-RELATED10 (MUCI10; Voiniciuc et al., 2015b) maintains the density of mucilage polymers and the structure of cellulose. In addition, highly branched xylan, elongated by IRREGULAR XYLEM14 (IRX14; Voiniciuc et al., 2015a; Hu et al., 2016) and substituted by MUCI21 (Voiniciuc et al., 2015a), is critical for mucilage attachment, and may link pectic polysaccharides to cellulosic rays. *In vitro* binding assays indicate that RG I chains can adhere to cellulose microfibrils via xylans (Ralet et al., 2016). Despite recent advances, additional enzymes that are required to determine the final structures of the polysaccharides detected in mucilage remain to be identified (Voiniciuc et al., 2015c).

In the past 15 years, several strategies have been successfully employed to discover genes involved in seed coat cell wall biogenesis (North et al., 2014). Forward genetic screens of mucilage-defective seeds in chemically mutagenized populations (Western et al., 2001, 2004; Dean et al., 2007; Arsovski et al., 2009; Huang et al., 2011; Voiniciuc et al., 2013), and natural Arabidopsis variants (Macquet et al., 2007; Saez-Aguayo et al., 2013) have yielded some of the key regulators of mucilage production and modification. Nevertheless, these screens likely have not been saturated since reverse genetic approaches based on seed coat transcriptional datasets have recently identified multiple glycosyltransferases directly involved in cell wall polysaccharide biosynthesis (Kong et al., 2013; Yu et al., 2014; Voiniciuc et al., 2015a,b; Hu et al., 2016).

To date, only two genes that affect the structure of mucilage polysaccharides were discovered based on the analysis of natural variants (North et al., 2014). Shahdara seeds from Tajikistan fail to release mucilage and float on water as result of defects in the MUCILAGE-MODIFIED2 (MUM2) β-galactosidase (Macquet et al., 2007), which trims galactan side chains from RG I (Dean et al., 2007). The Djarly accession from Kyrgyzstan also fails to release mucilage when imbibed in RR, due to a truncated version of PECTIN METHYLESTERASE INHIBITOR6 (PMEI6), a regulator of pectin modification (Saez-Aguayo et al., 2013). Four other accessions that have floating seeds despite mucilage release have also been isolated, but the causal mutations remain to be identified (Saez-Aguayo et al., 2014). Via an independent screen of RR-stained seeds, we identified around 50 additional accessions with clearly altered mucilage capsules. Three of these natural variants were recently shown to be heteromannan-deficient, unlike the Col-0 reference, based on immunolabeling of mucilage capsules with the LM21 monoclonal antibody (Voiniciuc et al., 2015b). The Lm-2 (Le Mans, France), Ri-0 (Richmond, Canada), and Lc-0 (Loch Ness, United Kingdom) accessions phenocopied the GGM-deficient *muci10* and *csla2* T-DNA insertion mutants with regards to seed coat morphology and mucilage phenotypes (Voiniciuc et al., 2015b). In this study, we describe in greater detail the altered mucilage phenotypes of 25 Arabidopsis accessions, including Lm-2, Ri-0, and Lc-0. Our results suggest that changes in the transcriptional regulation of *MUCI10* may contribute to the natural variation of Arabidopsis seed mucilage structure.

## Materials and methods

### Plant growth

The seeds of natural accessions were obtained from the Versailles Arabidopsis Stock Center (http://publiclines.versailles.inra.fr/naturalAccession/index). The original screen for accessions with impaired mucilage staining (Supplemental Table 3) was performed using seeds produced in a growth chamber as previously described (Saez-Aguayo et al., 2013) with a 16 h photoperiod at 21°C and 8 h dark at 18°C, 65% relative humidity and 170 μmol m^−2^ s^−1^. Plants were grown in compost (Tref Substrates) in individual 6 cm^2^ pots and watered with Plan-Prod nutritive solutions (Fertil). For all other experiments, plants were grown as previously described (Voiniciuc et al., 2015b,c) in individual round pots (Ø 5 cm; 35 multi-well inserts per tray) at constant light (around 170 μE m^−2^ s^−1^), temperature (20°C) and relative humidity (60%). Each plant was contained within an Aracon tube (Betatech bvba, http://www.arasystem.com), and seeds were harvested by shaking stems with mature, dry siliques into large paper bags.

### RR staining and area measurements

**Figure 1:**
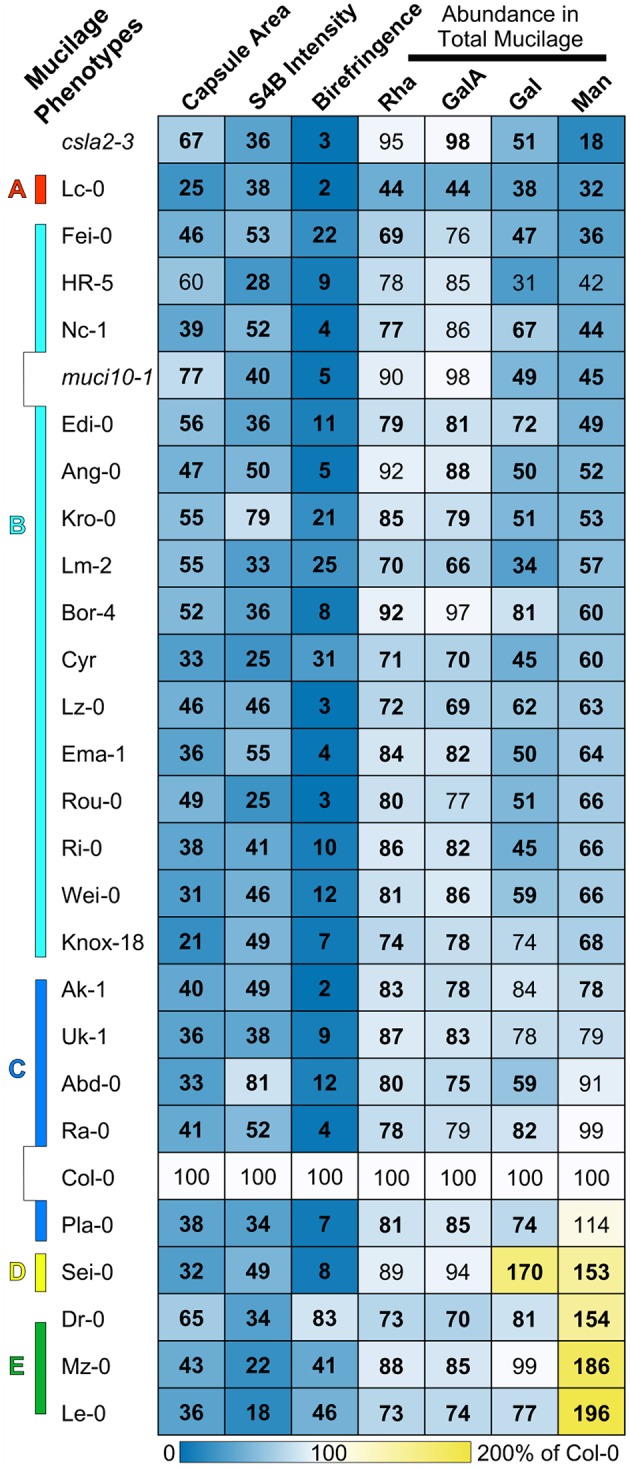
**Arabidopsis natural accessions display a wide range of mucilage defects**. Heatmap of mucilage traits analyzed with different techniques. Light microscopy was used to quantify the area of RR-stained mucilage capsules, S4B-labeled cellulose, and the birefringence of crystalline structures. The rows are sorted based on the content of Man, which was determined via monosaccharide analysis of total mucilage extracts. The mean value of each phenotype is expressed as a percent of the Col-0 reference (see calibration scale), and significant changes (*t*-test, *P* < 0.05) are shown in boldface. The 25 mucilage-modified accessions were classified into five groups **(A–E)** based on their phenotypes. The *csla2-3* and *muci10-1* T-DNA insertion mutants are deficient in GGM.

Using 24-well plates, 20–30 seeds were mixed with 500 μL of water for 5 min. After removing the water, mucilage was stained with 300 μL of 0.01% (w/v) RR (VWR International, A3488.0001) for 5 min. The dye solution was then replaced with 300 μL of water, and an image of each well was captured with a Leica MZ12 stereomicroscope equipped with a Leica DFC 295 camera. Seed and mucilage areas were quantified using the Fiji image processing software (Schindelin et al., 2012), as previously described (Voiniciuc et al., 2015b). Mucilage plus seed regions were segmented using the following color threshold (minimum, maximum) parameters: red (0, 255), green (0, 115), and blue (0, 255), while seeds were segmented using red (0, 120), green (0, 255), and blue (0, 255). Areas were measured with the Analyze Particles function (circularity = 0.5–1.0), excluding edges and extreme sizes (Supplemental Table 1). At least 10 seeds in each well passed all the selection criteria, and a total of more than 1500 seeds were quantified for Figure 1, Supplemental Table 1. Three biological replicates were analyzed per genotype, except only one for the HR-5 accession.

### S4B staining and intensity measurements

Water-hydrated seeds were stained with 0.01% (w/v) S4B (Sigma-Aldrich, 212490-50G) in 50 mM NaCl solution, exactly as previously described (Voiniciuc et al., 2015a). Fluorescent signals were detected with the Leica SP8 confocal system (552 nm excitation, 600–650 nm emission). S4B intensity across the seed surface was measured using the Analyze/Plot Profile function in Fiji. Straight lines (width of 200; covering 4–5 epidermal cells) were drawn perpendicular to the seed surface (Figure 1Q), and the resulting intensity plots were exported to Microsoft Excel. Two distinct sets of seed coat epidermal cells from a representative seed were measured per genotype. The S4B intensity values in Figure 1A represent the mean area under the intensity plots calculated using the trapezoidal rule (http://people.oregonstate.edu/~haggertr/487/integrate.htm) relative to Col-0.

### Quantification of mucilage birefringence

To visualize the birefringence of crystalline structures in mucilage (Voiniciuc et al., 2015b), around 20 water-hydrated seeds were transferred to cavity slides (VWR International, 631–9475), and were examined using plane polarized light on a Zeiss Axioplan2 microscope with a Zeiss AxioCam ICc 5 camera. The imaging was performed as described by the microscopy facility at the Icahn School of Medicine at Mount Sinai (http://icahn.mssm.edu/research/resources/shared-resource-facilities/microscopy/user-protocols). The relative amount of birefringence was quantified using a modified version of the Fiji macro commands used for the RR-stained mucilage area measurements. Birefringent regions were selected using the following color threshold (minimum, maximum) parameters: red (55, 255), green (140, 255), and blue (60, 255), and their areas were quantified using the Analyze Particles command (including holes, and summarizing the results). The total birefringent area in each image was divided by the number of seeds (at least 8; manually counted) to calculate the relative amount of birefringence per seed. Figure 1A shows the mean level of birefringence per seed (normalized to Col-0) of two sets of seeds (imaged on different days) harvested from the same plants.

### Mucilage monosaccharide composition

**Figure 2:**
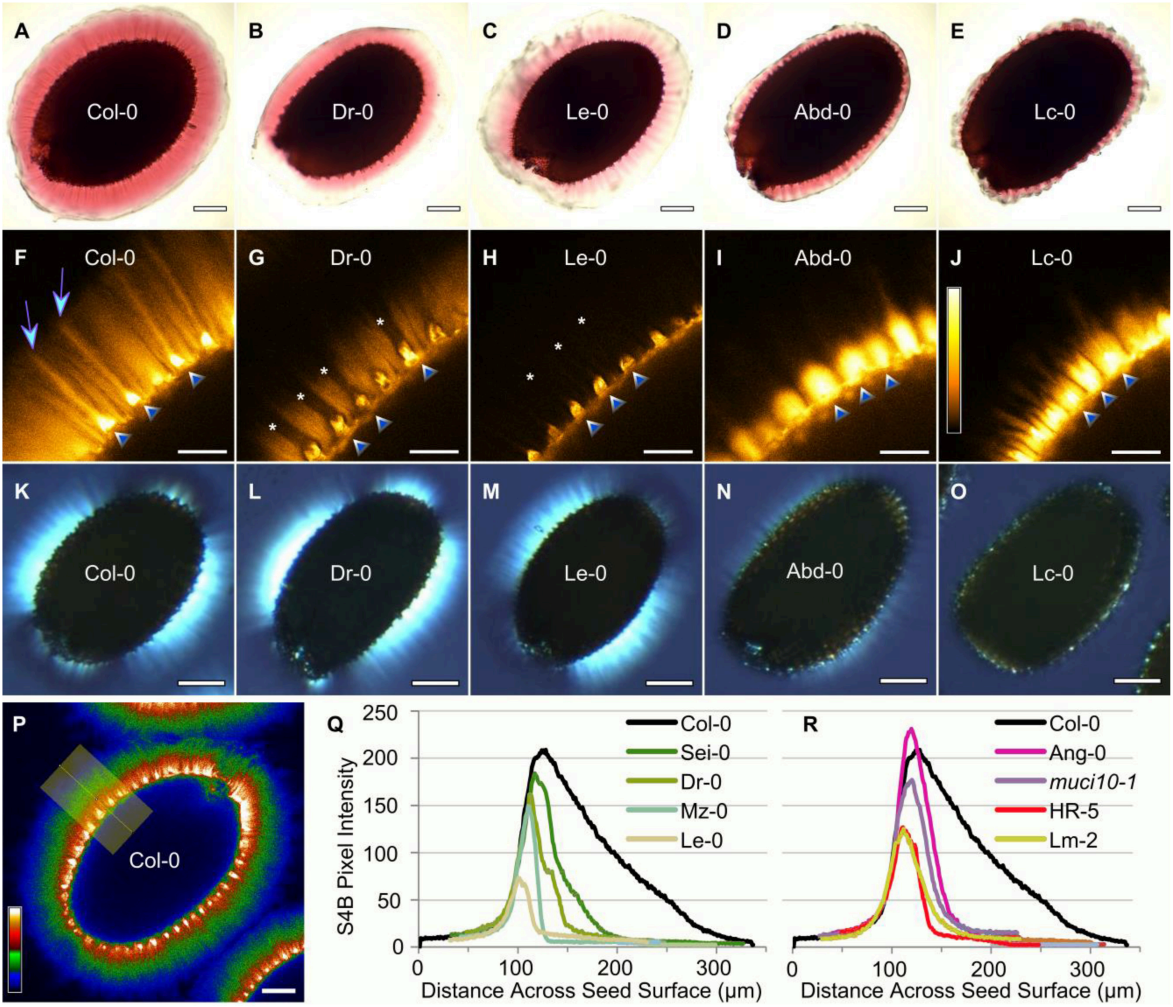
**Altered distribution of pectin, cellulose and crystalline structures**. Representative images for five of the accessions analyzed in Figure 1. RR-staining of adherent mucilage **(A–E)**, S4B labeling of cellulose **(F–J)**, and the birefringence of crystalline structures **(K–O)**. S4B signals were visualized with Orange Hot **(H–L)**, or Thal **(Q)** look-up tables in Fiji. Cellulosic rays are present in Col-0 (arrows) above columellae (triangles), but are absent in certain lines (asterisks). Based on S4B intensity across the seed surface **(P)**, increased **(Q)**, or decreased **(R)** mucilage Man content reduced cellulose distribution relative to Col-0. Plots were manually aligned, and the highest peaks represent the regions marked by triangles in **(F–J)**. Scale bars = 200 μm **(A–E,P)**; 50 μm **(F–J)**; 250 μm **(K–L)**.

The monosaccharide composition of mucilage was determined according to a protocol that has been described in great detail (Voiniciuc and Günl, 2016). Total mucilage was extracted by vigorously mixing 5 mg of seeds with 1 mL of water (containing 30 μg of ribose as internal standard) using a ball mill, operated for 30 min at 30 Hz. After the seeds settled at the bottom of each tube, 800 μL of each supernatant was transferred to a screw-cap tube, and dried under pressurized air at 45°C. Matrix polysaccharides were hydrolyzed using 300 μL of 2 M trifluoroacetic acid for 60 min at 120°C. After a final drying step, the monosaccharides were eluted in 600 μL of water, and quantified by high-performance anion-exchange chromatography with pulsed amperometric detection (HPAEC-PAD). A serial dilution of a nine-sugar mixture (Fucose, Fuc; Rhamnose, Rha; Arabinose, Ara; Galactose, Gal; Glucose, Glc; Xylose, Xyl; Mannose, Man; Galacturonic Acid, GalA; Glucuronic Acid, GlcA; all obtained from Sigma-Aldrich) was prepared alongside the unknown samples. HPAEC-PAD was performed using CarboPac PA20 guard (Dionex Softron, 060144) and analytical (Dionex Softron, 60142) columns on a Dionex DX-600 system equipped with AS50, GP50, ED50 modules. Since a maximum of 48 samples could be processed in parallel, the mucilage composition of the 25 accessions shown in Figure 2 was analyzed in three separate batches. Extracts from the Col-0 reference were prepared for each experiment and used for normalization (Supplemental Table 2).

### Analyses of genome sequences

Only 175 of the Arabidopsis natural accessions screened for defects in RR-stained mucilage had available genotyping data (Cao et al., 2011; Horton et al., 2012). Each accession was assigned a qualitative score for its RR-stained mucilage area relative to Col-0 capsules: larger (1.2), similar (1.0), partly smaller (0.8), moderately smaller (0.6), or very small (0.4). The complete phenotypic dataset is compiled in Supplemental Tables 3A,B. We analyzed these semi-quantitative values together with the 250 k single-nucleotide polymorphism (SNP) chip data of these lines (Horton et al., 2012). First, SNPs with a minor allele frequency of less than 0.05 were removed and the resulting data were analyzed with Factored Spectrally Transformed Linear Mixed Models (FaST-LMM) version 2.07 using “exact” inference (Lippert et al., 2011), since this set of tools has been shown to provide excellent statistical data and was developed to remove confounding effects such as population stucture. We also performed a genome-wide association study (GWAS) using the GWAPP tool (http://gwapp.gmi.oeaw.ac.at; Seren et al., 2012), which highlighted similar regions.

Polymorphisms in the *MUCI10* (At2g22900) sequence were analyzed for 23 accessions using the Arabidopsis 1001 Genome Browser (http://signal.salk.edu/atg1001/3.0/gebrowser.php). The preliminary genome sequencing data was provided by multiple groups at the Salk Institute, the Max Planck Institute for Developmental Biology, the Gregor Mendel Institute, the DOE Joint Genome Institute and the Monsanto Company (Cao et al., 2011; Gan et al., 2011). Nucleotide sequences (Chr2: 9743900-9748899) were exported from the browser, and converted to FASTA format using A Plasmid Editor (ApE; http://biologylabs.utah.edu/jorgensen/wayned/ape). Phylogenetic analysis was performed in MEGA6.0 (http://www.megasoftware.net; Tamura et al., 2013), according to a published guide (Hall, 2013). A Maximum Likelihood tree (Tamura 3-parameter model; Tamura, 1992) was built for 24 DNA sequences aligned with the MUSCLE method. Branches with less than 50% reliability (bootstrap method, 500 replicates) were condensed.

Transcription factor binding sites upstream of *MUCI10* were identified using the Plant Promoter Analysis Navigator (PlantPAN; http://PlantPAN2.itps.ncku.edu.tw; Chow et al., 2016), and cross-referenced with the peaks of the GWAS analysis and the sequence polymorphisms of the examined accessions in the Arabidopsis 1001 Genome Browser.

### Quantification of transcript levels

For each plant, three open flowers (0 d post anthesis, DPA) were marked with non-toxic paint to precisely select the stage of silique development (Dean et al., 2011). Total RNA was isolated from 7 DPA siliques (three per biological replicate) using the RNeasy Plant Mini Kit (Qiagen, 74904), according to the manufacturer's instructions. On-column digestion with RNase-Free DNase (Qiagen, 79254) was performed to remove any residual DNA. RNA concentration was measured using the Qubit RNA High Sensitivity Assay (Life Technologies) and 200 ng of each sample was used for cDNA synthesis with the iScript kit (BioRad, 170-8891) in a 20 μL reaction.

Primers for quantitative reverse transcription polymerase chain reaction (qRT-PCR) were designed using QuantPrime (http://www.quantprime.de; Arvidsson et al., 2008). *UBQ5* (AT3G62250; F: AAGAAGACTTACACCAAGCCGAAG; R: ACA GCGAGCTTAACCTTCTTATGC) and *elF4A1* (AT3G13920; F: ACCAACTTTGCTCCA GCATGGC; R: TGGTCAAACTGACGTGCATCAAAC) served as reference genes, due to their high expression stability (Gutierrez et al., 2008). *CSLA2* (F: ATTCCGTCGGTA CTCCAAGGTC; R: TAGCCCTTCCTGCCTCAAACAG) and *MUCI10* (F: TAC GCTGCGTTTCGTGAGGAAC; R: ACGAACGGTCTCCGTTTGCTTC) were target genes. The qRT-PCR was performed on a BioRad MyiQ system using 20 μL reactions containing iQ SYBR Green Supermix (BioRad), 300 nM of each primer and 0.2 μL of first-strand cDNA. For all genes, 40 amplification cycles were carried out at 60°C for annealing/extension. Negative controls without cDNA were included in every run. Primer amplification efficiencies were determined via a serial dilution of silique cDNA. Fold changes in target gene expression, normalized to the geometric mean of *UBQ5* and *elF4A1*, were calculated in Microsoft Excel (Pfaffl, 2001; Fraga et al., 2008).

### Transgene complementation

Arabidopsis natural accessions were grown in separate pots, and their first inflorescence shoots were removed. After 7 days, plants were transformed using a modified floral spray method (Weigel and Glazebrook, 2006). The generation of the *35S:MUCI10-sYFP* construct was previously described (Voiniciuc et al., 2015b). Plants were sprayed twice, 1 week apart, with an infiltration medium containing *Agrobacterium tumefaciens* GV3101::pMP90::pSOUP cells (with the *35S:MUCI10-sYFP* transgene), 5% (w/v) sucrose and 0.02% (v/v) Silwet L-77. Afterwards the plants were kept covered, for 24 h in the dark. Basta-resistant T_1_ seedlings were selected on soil by spraying a 10 mg/L glufosinate-ammonium solution (Bayer). Seedlings were resprayed every 2 days until most leaves turned yellow. The green leaves of Basta-resistant seedlings were screened for sYFP fluorescence on a Leica SP8 confocal microscope (488 nm excitation, 505–550 emission).

## Results

### Arabidopsis accessions show a wide range of mucilage staining defects

To discover how mucilage structure varies in *Arabidopsis thaliana* natural populations, we screened the seeds of around 280 accessions for defects in RR staining (full dataset in Supplemental Table 3). A surprisingly large number of lines (around 50 variants) released mucilage capsules noticeably different from the Col-0 reference accession. We further characterized only a subset of 25 variants (Table 1), which displayed smaller RR-stained capsules. These particular lines were selected since (in March 2014) they had been or were scheduled to be sequenced as part of the Arabidopsis 1001 Genomes Project (http://1001genomes.org; Weigel and Mott, 2009). The newly identified accessions with modified mucilage properties originate predominantly from Western and Central Europe, but also include two North American varieties: Ri-0 and Knox-18 (Knox, Indiana, USA).

**Table 1:** **Origins of the 25 Arabidopsis natural accessions described in this article**.

**AV #**	**Name**	**City**	**Country**	**Lat**.	**Long**.
28	Cyr	St. Cyr sur Loire	France	47.40	0.67
31	Lm-2	Le Mans	France	48.01	0.20
43	Sei-0	Seis am Schlern	Italy	46.54	11.56
51	Dr-0	Dresden	Germany	51.06	13.74
82	Rou-0	Rouen	France	49.44	1.10
83	Edi-0	Edinburgh	UK	55.96	−3.19
87	Lz-0	Lezoux	France	45.83	3.38
88	Ra-0	Randan	France	46.02	3.36
97	Le-0	Leiden	Netherlands	52.16	4.49
99	Ang-0	Angleur	Belgium	50.61	5.60
113	Uk-1	Umkirch	Germany	48.03	7.76
115	Ak-1	Achkarren	Germany	48.07	7.63
138	Mz-0	Merzhausen	Germany	47.97	7.83
160	Ri-0	Richmond	Canada	49.18	−123.13
171	Lc-0	Loch Ness	UK	57.34	−4.42
177	Abd-0	Aberdeen	UK	57.15	−2.22
195	Ema-1	East Malling	UK	51.29	0.45
210	Kro-0	Krotzenburg	Germany	50.08	8.97
218	Nc-1	Ville-en-Vermois	France	48.62	6.25
242	Wei-0	Weiningen	Switzerland	47.25	8.26
363	Knox-18	Knox (Indiana)	USA	41.28	−86.62
387	Bor-4	Borky	Czech Rep.	49.40	16.23
392	HR-5	Ascot	UK	51.40	−0.64
441	Fei-0	St. Maria D. Feiria	Portugal	40.92	−8.54
501	Pla-0	Playa de Aro	Spain	41.50	2.25

*This table shows the number of each accession (AV #) in the Versailles Arabidopsis Stock Center, and the location from which the seeds were first collected. Latitude (Lat.) and longitude (Long.) coordinates are expressed in degrees. Only Cyr, Knox-18, Bor-4, and HR-5 accessions have reliable GPS coordinates, while the others are only estimates*.

The accessions were regrown in the conditions previously used to screen *mucilage-related* mutants (Voiniciuc et al., 2015b), and showed heritable defects in RR staining. Compared to the Col-0 reference, the selected variants had 35–79% smaller RR-stained capsules (Figure 1). In contrast to their altered mucilage, these genotypes did not have any significant changes in seed area relative to Col-0 (*t*-test, *P* < 0.05), except for the 24% larger seeds of Lc-0 (Loch Ness, UK; Supplemental Table 1).

In addition to impaired RR staining of pectic polymers, we used light microscopy to observe two other mucilage properties that typically reflect the structure of cellulose. We developed custom macros in the Fiji image processing software for high-throughput quantification of the intensity of S4B fluorescence and the area of birefringent regions (containing crystalline polymers) around seeds. Relative to Col-0, accessions with modified RR staining also displayed significantly reduced S4B staining and smaller birefringent regions (Figure 1; Supplemental Figures 1, 2). In general, moderate reductions (at least 45% for 22 accessions) in S4B intensity correlated with severely smaller birefringent regions (of at least 88% for 17 variants). However, a few outliers lacked proportional decreases in S4B and birefringence levels (Figure 2), which were expected to reflect the structure of the same mucilage polymer (crystalline cellulose). For instance, Dr-0 (Dresden, Germany) had 83% of Col-0 birefringence levels, but only 34% S4B intensity. Although its defects were more severe, Le-0 (Leiden, Netherlands) showed a similar trend in its birefringence (46%) and S4B (18%) levels relative to Col-0. A closer look at the pattern of S4B staining around seeds revealed partial (Dr-0) or complete (Le-0) loss of the ray-like structures characteristic of Col-0 mucilage (Figures 2F–H). The seeds of other accessions, such as Abd-0 (Aberdeen, UK) and Lc-0, displayed very small RR-stained capsules and almost no birefringence, despite retaining bright S4B staining (Figure 2).

### Gal and Man abundance vary widely in mucilage-modified accessions

Cell wall modifications in the selected accessions were further quantified via HPAEC-PAD analysis of monosaccharides in total mucilage extracted by vigorously shaking seeds in water. This fast yet robust method was previously shown to reveal changes even in low-abundance hemicellulosic polysaccharides (Voiniciuc et al., 2015a,b). Rha and GalA (the backbone of RG I) typically represented around 90% of the total mucilage extracts (Supplemental Table 2), similar to other procedures that employed time-consuming dialysis or ethanol precipitation steps (Voiniciuc et al., 2015c). While most of the 25 mucilage-modified accessions produced only 10–30% less mucilage than Col-0, the Lc-0 variant had more severe reductions (56%) in Rha and GalA (Figure 1; Supplemental Table 2).

Previous mucilage immunolabeling experiments indicate that Lm-2, Ri-0, and Lc-0 are deficient in GGM polymers (Voiniciuc et al., 2015b), which consist of Gal, Glc, and Man subunits. Relative to Col-0, the total mucilage extracts of these three variants and most other accessions in Table 1 were found to primarily lack Gal and/or Man (Figure 1). In contrast, the absolute amount of Man was significantly increased (53–96%) in Dr-0, Le-0, Mz-0 (Merzhausen, Germany), and Sei-0 (Seis am Schlern, Italy). Surprisingly, only one of these four accessions (Sei-0) also produced more Gal (Figure 1). While altered GGM abundance should result in proportional changes in Glc and Man, 16 mucilage-modified accessions had inconsistent spikes in Glc (coefficient of variation above 0.4), which were not observed for other mucilage components (Supplemental Table 2). Glc represented only 1% of Col-0 mucilage extracts, but was at least 5 times more abundant in 23 of the 137 mucilage samples analyzed for Supplemental Table 2. These dramatic increases in Glc did not strongly correlate with the genotype and were not detected in other growth batches, while the changes in mucilage Gal and/or Man levels were stable for most accessions (data not shown).

Known mucilage mutants that disrupt the synthesis of different polysaccharides were grown and analyzed alongside the natural accessions. Consistent with previous results (Voiniciuc et al., 2015b), the GGM-deficient *muci10-1* and *csla2-3* lines had similar reductions in Gal (around 50%) compared to wild-type Col-0 mucilage (Figure 1), but contained distinct amounts of Man (45 vs. 18%). Most of the mucilage of Gal-deficient accessions more closely resembled the Man content of the *muci10-1* mutant, rather than *csla2-3* (Figure 1). Since altered GGM and xylan structures in mucilage have different effects on cellulose staining with S4B (Voiniciuc et al., 2015a,b), we also investigated if the accessions resembled the cellulose structure of hemicellulose-deficient mutants. Based on the intensity of S4B fluorescence across the seed surface, the cellulose distribution in both low-Man and high-Man accessions was similar to known GGM-deficient mutants (Figures 2P–R). The accessions did not have Xyl (Supplemental Table 2), S4B (Supplemental Figure 1), or birefringence (Supplemental Figure 2) levels consistent with the *muci21-1* and *irx14-2* xylan mutants (Voiniciuc et al., 2015a).

### Gwas analysis links *MUCI10* to mucilage staining defects

**Figure 3:**
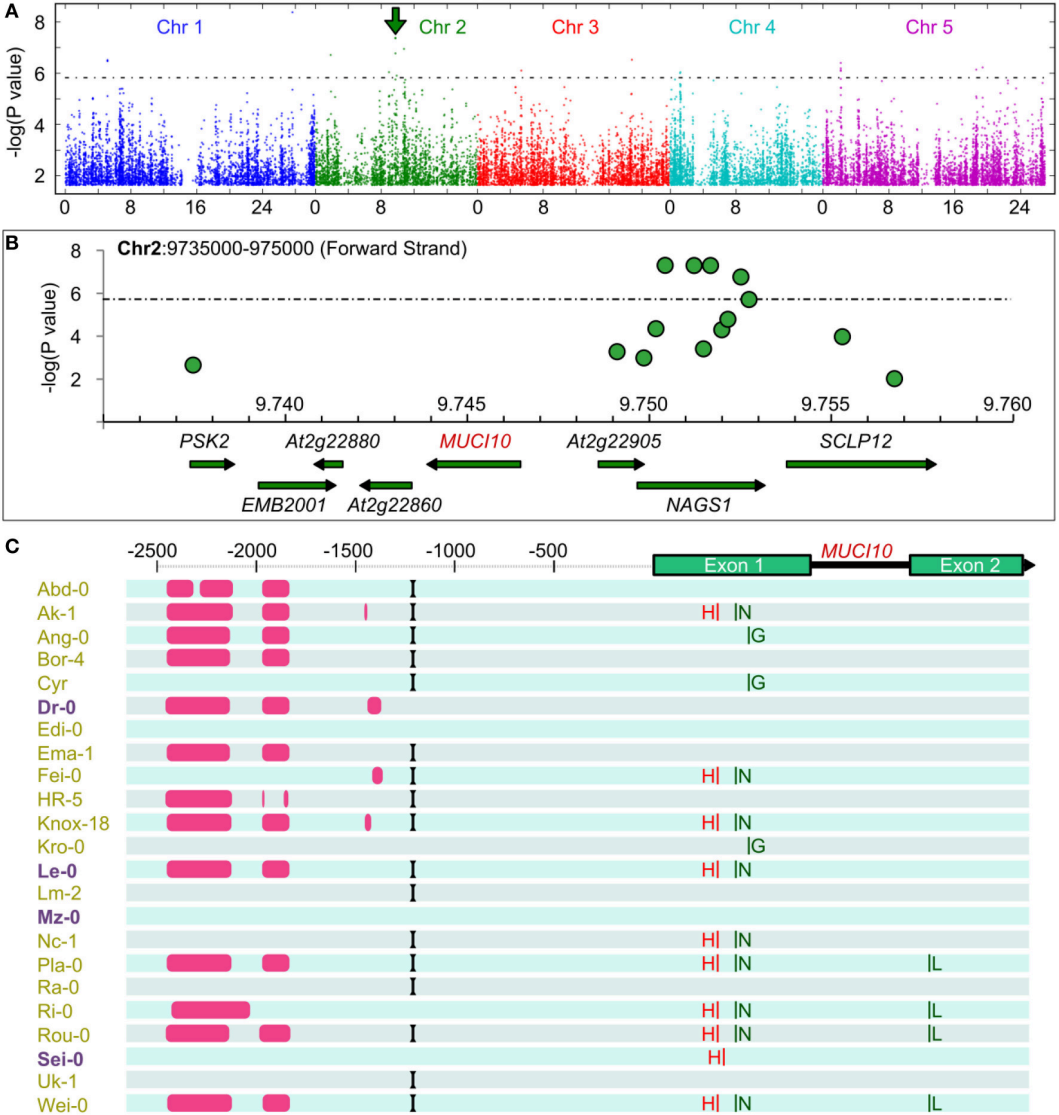
**Genome-wide association study links ***MUCI10*** to mucilage staining defects. (A)** Associations between SNPs and qualitative RR staining traits of 175 Arabidopsis natural accessions. Manhattan plots created using GWAPP (KW analysis, mac > 15, Seren et al., 2012) show color-coded chromosomes (Chr) and the 5% false discovery rate threshold (dashed line). The green arrow marks an interesting peak on Chr2. **(B)** The highest SNPs on Chr2 are near *MUCI10*. **(C)** Synonymous (green) and non-synonymous (red) amino acid changes relative to Col-0. Gaps (pink), an insertion (black), and many SNPs (not shown) were detected upstream of *MUCI10* in the Arabidopsis 1001 Genome Browser. Variants in purple are have more Man than Col-0.

Thanks to its ability to self-fertilize, Arabidopsis is particularly well suited to GWAS and more than a thousand different accessions have been genotyped (Korte and Farlow, 2013). Data from a 250K SNP chip (Horton et al., 2012) was available for 175 of the natural variants that we initially screened for semi-quantitative changes in RR-stained mucilage capsule size (Supplemental Table 3A). Since linear mixed models (LMM) are becoming the method of choice to correct for population structure and relatedness (Eu-ahsunthornwattana et al., 2014), we first performed GWAS analysis using FaST-LMM (Supplemental Figure 3). As we obtained qualitatively similar results in the GWAPP web application (http://gwapp.gmi.oeaw.ac.at; Seren et al., 2012), which can be conveniently accessed by other users, we then used in this tool for further data visualization. Figure 3A shows a summary of the associations between SNPs in the natural accessions and their RR-stained mucilage phenotypes (Supplemental Table 3A). Table 2 lists the 21 SNPs that were above the 5% false discovery rate threshold of the multiple testing procedure (Benjamini and Yekutieli, 2001). Chromosome 1 (Chr1), Chr2 and Chr5 each contained a region with at least three nearby SNPs above the significance threshold. To predict candidates that may affect mucilage traits, we analyzed the annotated functions and the transcription profiles of genes located in proximity to each GWAS peak (only nearest genes shown in Table 2). Two genes with uncharacterized functions, At3g50620 and At5g06930, were adjacent to significant SNPs and were preferentially expressed in developing seed coats at the time of mucilage production (Supplemental Figure 4). In addition to these candidates, close examination of the highest peak on Chr2 (arrow in Figure 3A) revealed an association with *MUCI10* (Figure 3B), which directly affects GGM synthesis and mucilage structure (Voiniciuc et al., 2015b). In contrast to *MUCI10*, no *CSLA* or *MANNAN SYNTHESIS-RELATED* genes (Wang et al., 2012) were found within 1 million bases (representing 100 times the average linkage disequilibrium decay in Arabidopsis; Kim et al., 2007) of the GWAS peaks in Table 2. Although, *MUCI10* is the only cell wall-related gene near the Chr2 peak, two other genes are expressed in seeds according to the public microarray data (Winter et al., 2007; Belmonte et al., 2013). While At2g22870 (*EMBRYO DEFECTIVE 2001, EMB2001*) is primarily expressed in the embryo, At2g22910 (*NAGS1*), which is predicted to facilitate amino acid synthesis (Kalamaki et al., 2009) is expressed at low levels throughout seed coat development (Supplemental Figure 4). Since *MUCI10* was the only gene predicted by GWAS known to affect the synthesis of Man-containing polymers, and most of the mucilage-modified natural variants phenocopied the *muci10-1* mutant defects (Figures 1, 2), we focused on this promising candidate for further experiments.

**Table 2:** **Top SNPs from the GWAS analysis and the nearest genes**.

**SNP Position**		**−log10 *P*-value**	**Nearest gene**	**Araport11 annotated function**
Chr1	5144264	6.51	At1g14910	ENTH/ANTH/VHS superfamily protein
Chr1	5144469	6.47		
Chr1	5144653	6.47		
Chr1	27655299	8.37	At1g73580	Calcium-dependent lipid-binding (CaLB domain)
Chr2	1859413	6.70	At2g05160	CCCH-type zinc finger family protein
Chr2	8973773	6.04	At2g20840	Secretory carrier membrane protein (SCAMP3)
Chr2	9750472	7.36	At2g22910	N-acetyl-l-glutamate synthase 1 (NAGS1)
Chr2	9751265	7.36		
Chr2	9751714	7.36		
Chr2	9752535	6.77		
Chr2	10799538	6.94	At2g25350	Phox (PX) domain-containing protein
Chr3	5368453	6.10	At3g15880	WUS-interacting protein 2 (WSIP2)
Chr3	18794378	6.52	At3g50620	P-loop containing nucleoside triphosphate hydrolase
Chr4	1195946	5.99	At4g02710	Kinase interacting (KIP1-like) protein (NET1C)
Chr4	1223971	6.03	At4g02760	RNI-like superfamily protein
Chr5	2143203	6.4	At5g06920	FASCICLIN-like arabinogalactan protein 21 (FLA21)
Chr5	2143640	6.18		
Chr5	2143874	6.11		
Chr5	2145769	6.08	At5g06930	nucleolar-like protein
Chr5	18632173	6.14	At5g45950	GDSL-like Lipase/Acylhydrolase superfamily protein
Chr5	19410479	6.22	At5g47940	40S ribosomal protein S27

*List includes only SNPs above the 5% false discovery rate threshold (dashed line in Figure 3A), calculated in GWAPP using the multiple testing procedure (Benjamini and Yekutieli, 2001). The putative function of the gene closest to each SNP was obtained from Araport (Krishnakumar et al., 2015)*.

**Figure 4:**
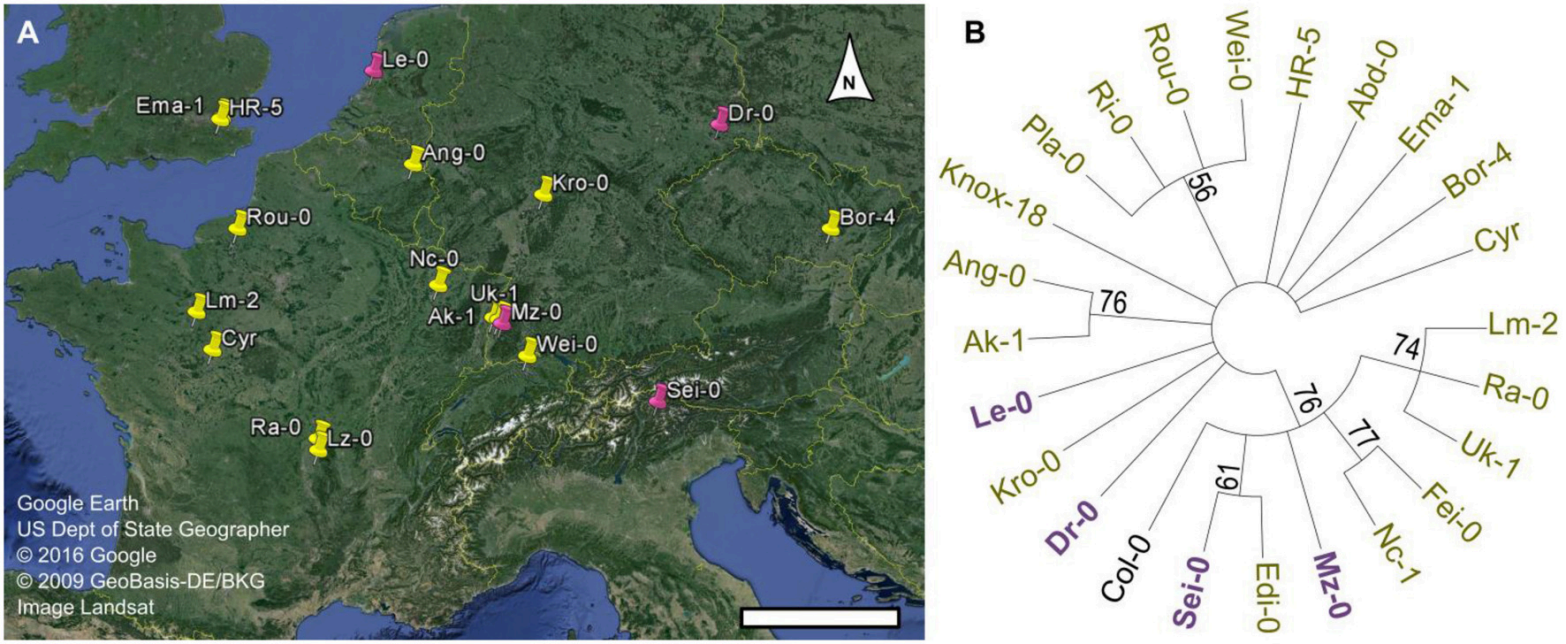
**Geographical distribution and phylogenetic analysis of Man-rich accessions. (A)** Map of Europe exported from Google Earth Pro. Arabidopsis natural accessions rich in Man originate from the pink sites, while GGM-deficient variants were collected from the locations marked in yellow. Scale bar = 400 km. **(B)** Phylogenetic analysis of *MUCI10* coding and upstream sequences (Chr2: 9743900-9748899). A condensed tree (50% cut-off) was built in MEGA6.0 (Tamura et al., 2013), and branch reliability was tested via the bootstrap method (500 replicates).

The Arabidopsis 1001 Genome Browser (http://signal.salk.edu/atg1001/3.0/gebrowser.php) was then used to compare the nucleotide sequence of *MUCI10* in Col-0 and 23 mucilage-modified accessions (Figure 3C). Sei-0 had a P119H substitution (Proline at position 119 changed to Histidine), while nine other accessions (including the Man-rich Le-0 variant) contained a non-synonymous SNP that induced R109H (marked with a red H in Figure 3C). Although, both changes result in amino acids with distinct chemical properties (Betts and Russell, 2007), these SNPs occur between the MUCI10 transmembrane and galactosyltransferase domains annotated in the ARAMMEMNON database (http://aramemnon.botanik.uni-koeln.de; Schwacke et al., 2003). While the SNPs in the *MUCI10* coding sequence do not have obvious deleterious effects, the preliminary 1001 Genome data suggests that many of the mucilage-modified accessions have large gaps (see pink bars in Figure 3C) and other polymorphisms in the large intergenic region upstream of the *MUCI10* start codon. We examined if the *MUCI10* polymorphisms correlate with the mucilage chemotypes reported in Figure 1, but did not identify sets of mutations that were consistent with the phenotypes. Indeed, the Man-rich accessions (Dr-0, Le-0, Mz-0, and Sei-0), which were collected from distinct parts of Europe (Figure 4A), clustered with Man-deficient accessions in a phylogenetic tree of *MUCI10* coding and upstream sequences (Figure 4B).

### Man-deficient accessions have reduced expression of *MUCI10*, not *CSLA2*

Based on the biochemical profiling (Figure 1) and the GWAS results (Figure 3), we hypothesized that altered expression of *MUCI10* may contribute to natural variation in mucilage structure. The Plant Promoter Analysis Navigator (PlantPAN; http://PlantPAN2.itps.ncku.edu.tw; Chow et al., 2016) was used to identify transcription factor binding sites upstream of *MUCI10*. We filtered a large list of putative regulators of *MUCI10* based on their proximity to the high GWAS peaks (Figure 3A, Table 2). Only nine of transcription factors had conserved motifs upstream of *MUCI10* that were affected by the large sequencing gaps or other genetic polymorphisms in at least one of the accessions (Supplemental Table 4). One of these candidates (At2g22800), which encodes the homeobox protein HAT9, was nearby *MUCI10* (38075 bp away) and was also up-regulated in the seed coat at the developmental stage of secondary cell wall production (Supplemental Figure 4).

**Figure 5:**
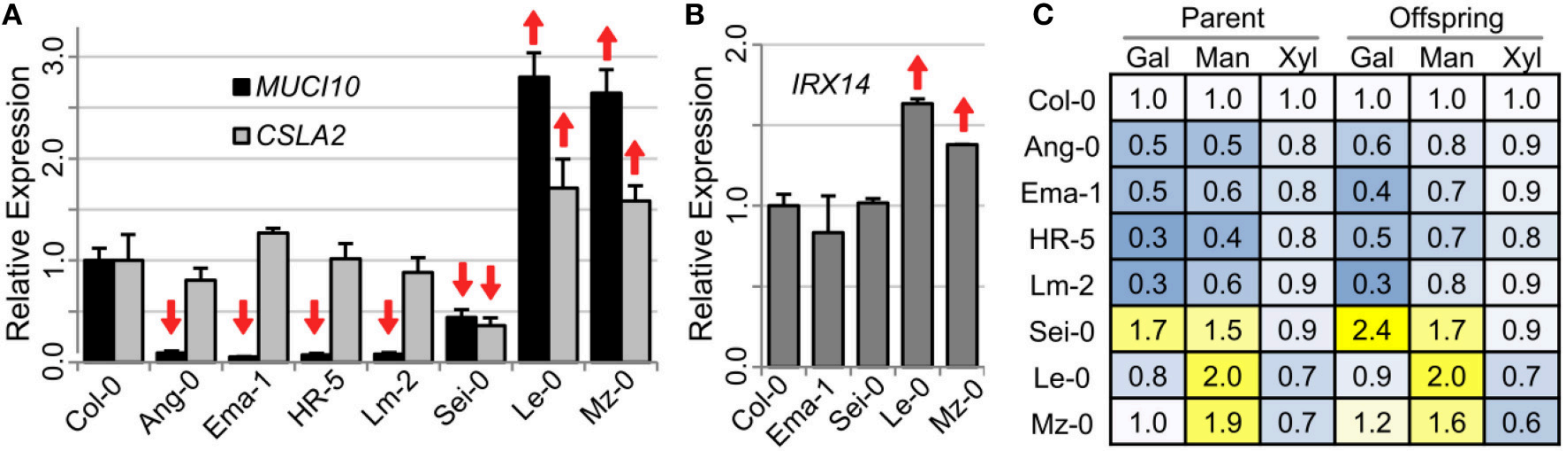
**Gene expression in siliques of accessions with altered Gal and Man content. (A,B)** qRT-PCR analysis of RNA isolated from 7 DPA siliques. Data show means + SD of two biological replicates analyzed at least twice (only technical error shown for one Le-0 plant). Target gene transcript levels were first normalized to the geometric mean of two reference genes (*UBQ5* and *elF4a*), and were then set as 1.0 for Col-0. Significant changes in gene expression relative to Col-0 (*t*-test, *P* < 0.05) are indicated up or down arrows. **(C)** Heatmap of mucilage sugars (Col-0 levels set as 1.0) from two sequential generations. Gal and Man levels are altered more than the content of Xyl.

Using RNA isolated from 7 DPA siliques, we performed qRT-PCR to investigate if six mucilage-modified accessions have altered expression of *MUCI10* or *CSLA2* (Figure 5), which are both required for GGM synthesis (Voiniciuc et al., 2015b). Ang-0 (Angleur, Belgium), Ema-1 (East Malling, UK), HR-5 (Ascot, UK), and Lm-2 had at least 90% fewer *MUCI10* transcripts compared to Col-0 (Figure 5A), and were deficient in Gal and Man sugars (Figure 5C). Consistent with their closer resemblance to the mucilage chemotype of *muci10* rather than *csla2* (Figure 1), the low-Man variants had no significant changes in *CSLA2* expression (Figure 5A).

Three additional variants (Sei-0, Le-0, and Mz-0) were selected for qRT-PCR analysis due to their high Man content (Figure 5C). Le-0 and Mz-0 had significant increases in *MUCI10* and *CSLA2* expression levels compared to Col-0 (Figure 5A). Although Sei-0 produced consistently more Gal and Man than Col-0 (Figure 5C), it had significantly lower expression of both *MUCI10* and *CSLA2* (56% and 66%, respectively). To check if these accessions specifically affect GGM-related genes or hemicellulose biosynthesis in general, we also analyzed the expression of *IRX14*, which is the critical for the elongation of xylan polymers in seed mucilage (Voiniciuc et al., 2015a). While Col-0, Ema-1 and Sei-0 siliques had similar *IRX14* transcript levels, Le-0 and Mz-0 showed higher xylan gene expression (Figure 5B). This suggests that Sei-0 has a specific down-regulation of GGM-related genes, but Le-0 and Mz-0 have broader transcriptional changes that also affect xylan synthesis.

### *MUCI10* overexpression rescues Ema-1 GGM-deficiency and RR staining defects

**Figure 6:**
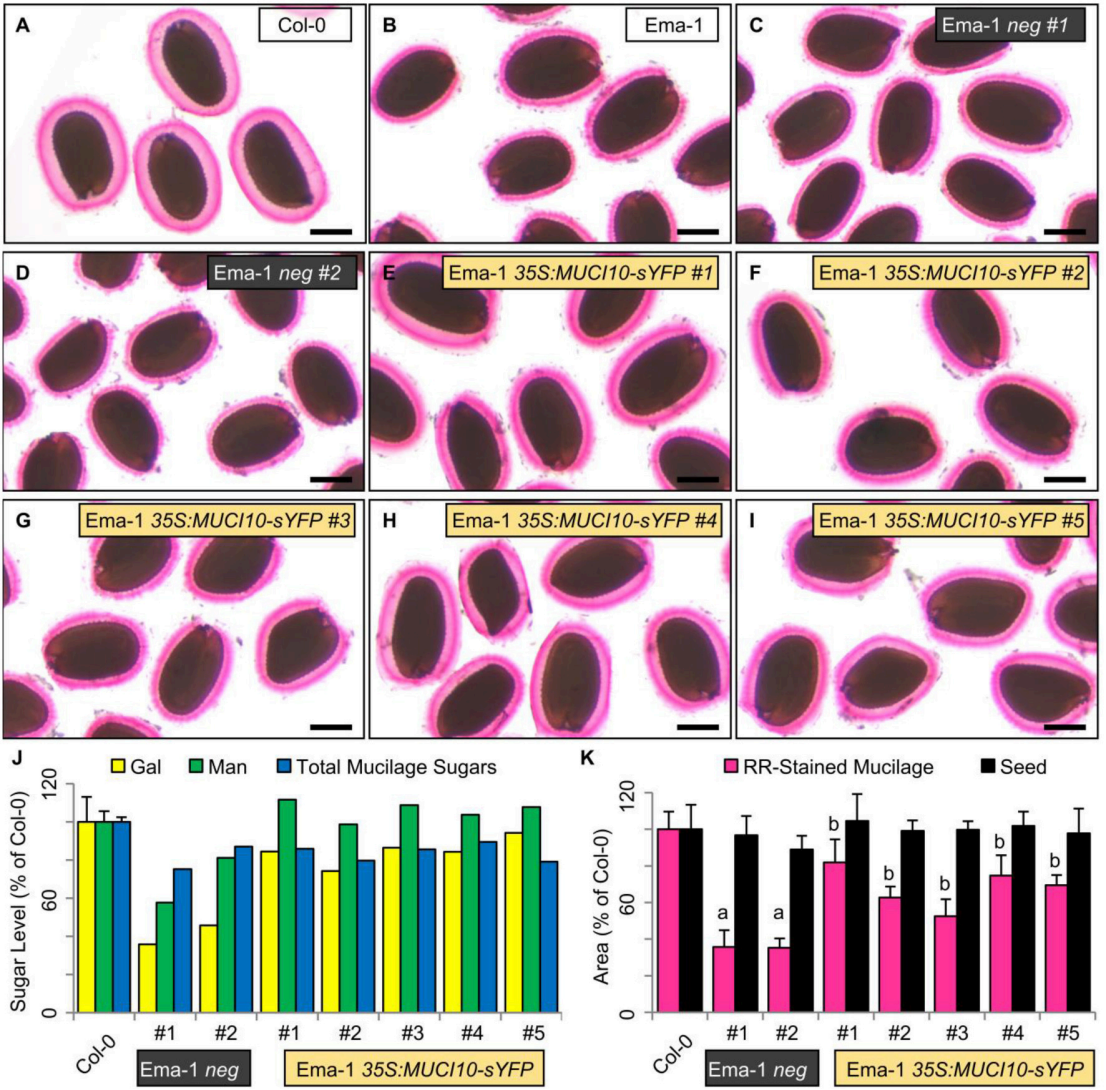
*****35S:MUCI10-sYFP*** transgene complementation of Ema-1 mucilage structure. (A–I)** RR mucilage staining phenotypes of Col-0, Ema-1, and T1 lines with or without MUCI10-sYFP expression. Two Ema-1 *neg* plants survived the Basta selection but did display any sYFP signal. Scale bars = 250 μm. **(J)** Gal, Man and total mucilage sugars amounts expressed as a percent of the Col-0 values. Data shows means + SD of four Col-0 biological replicates, and the seven independent T_1_ lines. **(K)** Mucilage and seed area expressed as a percent of the Col-0 values. Data show means + SD of more than five seeds. Significant changes relative to Col-0 (*t*-test, *P* < 10^−7^), or Ema-1 neg #1 dimensions (*t*-test, *P* < 10^−2^) are marked by “a” and “b”, respectively.

Since four accessions deficient in Gal and Man had decreased *MUCI10* transcription (Figure 5), potentially due to a missing transcription factor binding site (Supplemental Table 4), we tested if the constitutive expression of this gene could rescue the observed mucilage defects. We previously demonstrated that the *35S*-driven expression of MUCI10 tagged with yellow super fluorescent protein (*35S:MUCI10-sYFP*) could rescue the *muci10-1* T-DNA mutant defects, unlike the *35S:sYFP* control (Voiniciuc et al., 2015b). The flowers of multiple Man-deficient natural accessions (Lm-2, Ang-0, HR-5, Ri-0, and Ema-1) were therefore sprayed with *Agrobacterium* cells containing the functional *35S:MUCI10-sYFP* transgene. The resulting T_1_ seedlings were first selected for Basta resistance on soil, and were then screened for fluorescent MUCI10-sYFP punctae. Transgene complementation was only observed for Ema-1 (Figure 6; Supplemental Table 5), although fewer sYFP-expressing transformants were recovered for the other variants (3 for Lm–2, 1 for Ang-0, none for HR5, 1 for Ri-0). We identified five independent Ema-1 *35S:MUCI10-sYFP* T_1_ lines that displayed sYFP punctae and RR staining phenotypes more similar to Col-0 than Ema-1. Two other T_1_ plants (called Ema-1 *neg*) survived the Basta selection but did not show any MUCI10-sYFP fluorescence and produced 65% smaller mucilage capsules and 50% less Gal than Col-0 (Figures 6A,B), similar to the untransformed Ema-1 plants (Figures 1, 5C). In contrast to the negative controls, the mucilage Gal and Man amounts, as well as the mucilage capsule area were at least partially restored in five independent Ema-1 T_1_ lines that expressed *35S:MUCI10-sYFP* (Figure 6).

## Discussion

### Arabidopsis accessions show a wide range of mucilage defects

This study highlights the extensive natural variation of Arabidopsis seed mucilage architecture. We identified a surprisingly large number of accessions (mainly from Western Europe) that showed clear changes in the area of RR-stained mucilage compared to Col-0. Previously, only 14 accessions were reported to have altered mucilage, and they were all collected from Central Asia and Scandinavia (Macquet et al., 2007; Saez-Aguayo et al., 2013, 2014). These variants displayed seed flotation in water due to the absence of mucilage capsules around seeds. Despite vast changes in mucilage composition compared to the reference wild type, the 25 accessions analyzed in this study produce seeds that sink in water (Table 1). Based on the analysis of mucilage monosaccharide composition and imaging experiments, we classified the 25 natural variants into 5 phenotypic classes (marked by letters in Figure 1). The Arabidopsis “monster” from Loch Ness (Lc-0) was the only accession that produced larger seeds than Col-0, but only half as much pectin (phenotypic class A). While Lc-0 seed coat epidermal cells were not noticeably different from those of Col-0 in scanning electron micrographs (Voiniciuc et al., 2015b), the morphology of other cell types in these seeds remain to be investigated.

The second phenotypic class (B in Figure 1) has 15 members that closely resemble the Gal and Man amounts of *muci10-1* total mucilage extracts. Based on published immunolabeling experiments, at least two of these natural variants (Lm-2 and Ri-0) lack detectable heteromannan polysaccharides in seed mucilage (Voiniciuc et al., 2015b). This suggests that class B accessions have reduced content of GGM similar to the *muci10-1* mutant. Although, *csla2-3* and *muci10-1* insertion mutants were previously found to have proportional decreases in the content of Glc and Man in total mucilage extracts (Voiniciuc et al., 2015b), many of the mucilage samples analyzed in Supplemental Table 2 showed inconsistent spikes in Glc content that masked the expected changes. Since our procedure may also extract small molecular weight sugars, the Glc spikes are potentially contaminants that do not reflect changes in mucilage polysaccharides. While mucilage Gal and/or Man levels were stable for most accessions, dramatic increases in Glc levels (Supplemental Table 2) were not detected in other growth batches and did not generally correlate with the genotype. Therefore, heteromannan structure in the class B natural variants requires further examination. Nevertheless, accessions with at least 40% less Gal and/or Man displayed mucilage capsules with properties similar to known GGM mutants: more compact RR-stained capsules, severe reductions in S4B-stained cellulose and smaller birefringent areas relative to Col-0 (Figure 1; Voiniciuc et al., 2015b). Five additional variants (class C phenotype) had relatively minor changes in Gal and Man levels, but still severely disrupted mucilage properties in the imaging experiments (Figure 1). The class B and C accessions could be grouped more precisely in the future via the analysis of mucilage polysaccharides in greater detail.

Although, four accessions contained significantly more Man in mucilage extracts compared to Col-0, they had two distinct sets of mucilage phenotypes (see D and E, Figure 1). Only the Sei-0 accession had a proportional increase in Gal, and severely reduced birefringence (14% of Col-0 level). In contrast, Dr-0, Mz-0, and Le-0 had minor reductions or no change in Gal content. Despite showing 64–82% less S4B fluorescence (more severe than Sei-0; Figure 1), these three natural variants had only small decreases or no change in birefringence. S4B preferentially fluoresces in the presence of cellulose (Anderson et al., 2010), which is typically found in Arabidopsis seed mucilage in a crystalline form that causes birefringence of polarized light (Sullivan et al., 2011; Ben-Tov et al., 2015). Insertion mutants with severe mucilage detachment, such as *muci21-1* and *irx14-2*, display similar cellulose structures in birefringence and S4B analyses (Supplemental Figures 1, 2). The normal level of birefringence around Dr-0, Mz-0, and Le-0 seeds, therefore, diverged from previous observations. It is tempting to speculate that class E mucilage might contain larger amounts of another birefringent polysaccharide such as unbranched heteromannan, instead of cellulose. While heteromannans are likely synthesized in the Golgi in a highly substituted form, they can be trimmed in the cell wall by α-galactosidases (Scheller and Ulvskov, 2010). However, enzymes that remove heteromannan branches in Arabidopsis have yet to be described. In contrast to the highly branched GGM of Col-0 mucilage (Voiniciuc et al., 2015b), we hypothesize that class E accessions might contain larger amounts of unsubstituted glucomannan chains that form crystal structures via hydrogen bonding (Millane and Hendrixson, 1994). Increased Man content and proportionally less Gal in class E mucilage extracts is in accord with this hypothesis. Unlike the Glc spikes detected in other variants, increased Man levels in class D and E accessions were stably inherited (Figure 5C).

### *MUCI10* may contribute to natural variation in seed mucilage staining

The most significant SNP detected on Chr2 (the second highest overall; Table 2), in our GWAS analysis was located near *MUCI10* (Figures 3A,B). Polymorphisms in this gene could explain the class B mucilage phenotype (Figure 1), which includes 15 accessions identified in this study. Our analysis of preliminary sequencing data from the Arabidopsis 1001 Genome project did not identify SNPs likely to severely disrupt protein function (Figure 3C), but there were several large sequencing gaps upstream of the *MUCI10* start codon. Since we hypothesized that altered expression of *MUCI10* may contribute to natural variation in mucilage structure, we identified many putative regulators of this gene that are also near the GWAS peaks. By selecting only the cis-elements that are affected by the sequence gaps or other polymorphisms in at least one of the accessions, we predicted a shortlist of nine transcription factors that might target *MUCI10* (Supplemental Table 4). At2g22800 (HAT9) is up-regulated in the seed coat at the stage of secondary cell wall production (Supplemental Figure 4), and represents one of the more promising candidates that should be examined in future studies.

Four class B accessions (Ang-0, Ema-1, HR-5, and Lm-2) had at least 91% lower levels of *MUCI10* transcription, but did not disrupt the expression of *CSLA2* compared to the Col-0 reference (Figure 5A). In contrast, accessions with high-Man content showed more complex transcriptional changes in genes required for hemicellulose biosynthesis (Figure 5). Although, *MUCI10* was specifically down-regulated in the low-Man variants examined, we did not identify a consistent set of polymorphisms that correlated with the qRT-PCR results. Relative to Col-0 (Figure 5), *MUCI10* transcription was increased in Le-0 (large gaps in promoter sequence; Figure 3C) but decreased in Lm-2 (no gaps). This indicates that the large gaps reported in the Arabidopsis 1001 Genome Browser are unlikely to be the causal factor. The putative gaps are not necessarily deletions and might rather result from incomplete sequencing of this region in some accessions. In addition, the Edi-0 accession phenocopied the *muci10-1* insertion mutant (Figure 1), despite having no SNPs in the *MUCI10* coding sequence or insertions/deletions in the upstream region (Figure 3C). Since the low-Man and high-Man accessions clustered together in our phylogenetic analysis of *MUCI10* sequences (Figure 4B), the mutations that underlie the observed phenotypes likely reside elsewhere.

The modification of transcript levels for *MUCI10*, despite no consistent set of mutations in this locus, suggests that one of its transcriptional regulators might be disrupted in at least some of the Man-deficient natural accessions. Indeed, transformation of Ema-1 with a *35S:MUCI10-sYFP* transgene at least partially complemented its mucilage composition and RR staining defects (Figure 6; Supplemental Table 5). Five independent Ema-1 *35S:MUCI10-sYFP* T_1_ lines had mucilage Gal and Man amounts similar to Col-0, unlike the negative controls (Figure 6). Since Ema-1 did not contain any unique polymorphisms in *MUCI10*, we hypothesize that this accession may affect a relevant transcription factor. Constitutive expression of *MUCI10-sYFP* under the *35S* promoter would be able to complement variants missing a *MUCI10* activator, or overexpressing a *MUCI10* repressor.

### Mucilage-modified accessions are a valuable resource for cell wall research

This public collection of natural variants can be used to explore the architecture of seed mucilage and the biogenesis of its polysaccharide components. Since the accessions identified in this study fall into five phenotypic groups that are still loosely defined (Figure 1), future research should examine their cell wall defects using additional techniques such as linkage analysis and immunolabeling to elucidate their effects on polysaccharide structure. In addition, backcrosses to Col-0 will be necessary to establish the segregation pattern of each trait. This collection might reveal novel players that influence how seed coat epidermal cells produce an optimal amount of heteromannan, with a correct degree of galactosylation. Although, MUCI10 was already known to be involved in this process (Voiniciuc et al., 2015b), our GWAS analysis predicted two proteins of unknown function (At3g50620 and At5g06930) and several transcription factors that might affect cell wall structure. In addition to identifying novel cell wall-related genes, these accessions could also be exploited to investigate the functions of mucilage in nature. The ecological roles of Arabidopsis seed mucilage remain unclear and no association could be made between the mucilage phenotypic classes established here and the geolocalization of their collection site (Table 1, Figure 4A). It will be necessary to obtain more information about the collection sites in order to uncover potential links to the observed natural variation in mucilage characteristics. The imminent completion of the 1001 Genomes Project (http://1001genomes.org) and the availability of high-throughput techniques for the quantification of RR-stained pectin, S4B-labeled cellulose, and birefringent crystalline structures in mucilage will facilitate additional screens of natural accessions, and the mapping of genetic associations with higher precision.

## Author contributions

CV, HN, and BU conceived the initial screen, and CV performed it using seeds provided by HN. CV designed the other experiments, and EZ performed most of them. MS, MG assisted with analysis of monosaccharides. LF performed FaST-LMM tests. CV prepared figures, and wrote the paper.

## Funding

This work was supported by the Natural Sciences and Engineering Research Council of Canada (PGS-D3 grant to CV), the Ministry of Innovation, Science, and Research of North-Rhine Westphalia within the framework of the North-Rhine Westphalia Strategieprojekt BioEconomy Science Center (grant no. 313/323–400–00213 to MS and BU), and Saclay Plant Sciences (travel grant to CV).

### Conflict of interest statement

The authors declare that the research was conducted in the absence of any commercial or financial relationships that could be construed as a potential conflict of interest.

## References

[B1] Anderson C. T. Carroll A. Akhmetova L. Somerville C. R. (2010). Real-time imaging of cellulose reorientation during cell wall expansion in Arabidopsis roots. Plant Physiol. 152, 787–796. 10.1104/pp.109.150128 19965966 PMC2815888

[B2] Arsovski A. A. Popma T. M. Haughn G. W. Carpita N. C. Mccann M. C. Western T. L. (2009). AtBXL1 encodes a bifunctional beta-D-xylosidase/alpha-L-arabinofuranosidase required for pectic arabinan modification in Arabidopsis mucilage secretory cells. Plant Physiol. 150, 1219–1234. 10.1104/pp.109.138388 19458117 PMC2705025

[B3] Arvidsson S. Kwasniewski M. Riaño-Pachón D. M. Mueller-Roeber B. (2008). QuantPrime–a flexible tool for reliable high-throughput primer design for quantitative PCR. BMC Bioinformatics 9:465. 10.1186/1471-2105-9-465 18976492 PMC2612009

[B4] Belmonte M. F. Kirkbride R. C. Stone S. L. Pelletier J. M. Bui A. Q. Yeung E. C.. (2013). Comprehensive developmental profiles of gene activity in regions and subregions of the Arabidopsis seed. Proc. Natl. Acad. Sci. U.S.A. 110, E435–E444. 10.1073/pnas.1222061110 23319655 PMC3562769

[B5] Benjamini Y. Yekutieli D. (2001). The control of the false discovery rate in multiple testing under dependency. Ann. Stat. 29, 1165–1188. 10.1214/aos/1013699998

[B6] Ben-Tov D. Abraham Y. Stav S. Thompson K. Loraine A. Elbaum R.. (2015). COBRA-LIKE 2, a member of the GPI-anchored COBRA-LIKE family, plays a role in cellulose deposition in Arabidopsis seed coat mucilage secretory cells. Plant Physiol. 167, 711–724. 10.1104/pp.114.240671 25583925 PMC4347734

[B7] Betts M. J. Russell R. B. (2007). Amino-acid properties and consequences of substitutions, in Bioinformatics for Geneticist 2nd Edn., ed Barnes M. R. (Chichester: John Wiley & Sons, Ltd.), 311–342. 10.1002/9780470059180.ch13

[B8] Cao J. Schneeberger K. Ossowski S. Günther T. Bender S. Fitz J.. (2011). Whole-genome sequencing of multiple *Arabidopsis thaliana* populations. Nat. Genet. 43, 956–963. 10.1038/ng.911 21874002

[B9] Chow C.-N. Zheng H.-Q. Wu N.-Y. Chien C.-H. Huang H.-D. Lee T.-Y.. (2016). PlantPAN 2.0: an update of plant promoter analysis navigator for reconstructing transcriptional regulatory networks in plants. Nucleic Acids Res. 44, D1154–D1160. 10.1093/nar/gkv1035 26476450 PMC4702776

[B10] Dean G. H. Cao Y. Xiang D. Provart N. J. Ramsay L. Ahad A.. (2011). Analysis of gene expression patterns during seed coat development in Arabidopsis. Mol. Plant 4, 1074–1091. 10.1093/mp/ssr040 21653281

[B11] Dean G. H. Zheng H. Tewari J. Huang J. Young D. S. Hwang Y. T.. (2007). The Arabidopsis MUM2 gene encodes a beta-galactosidase required for the production of seed coat mucilage with correct hydration properties. Plant Cell 19, 4007–4021. 10.1105/tpc.107.050609 18165329 PMC2217648

[B12] Eu-ahsunthornwattana J. Miller E. N. Fakiola M. Jeronimo S. M. B. Blackwell J. M. Cordell H. J. (2014). Comparison of methods to account for relatedness in genome-wide association studies with family-based data. PLoS Genet 10:e1004445 10.1371/journal.pgen.1004445 25033443 PMC4102448

[B13] Fraga D. Meulia T. Fenster S. (2008). Real-time PCR, in Current Protocols Essential Laboratory Techniques 1st Edn., eds Gallagher S. R. Wiley E. A. (Hoboken, NJ: John Wiley & Sons, Inc.), 1–33. 10.1002/9780470089941.et1003s00

[B14] Gan X. Stegle O. Behr J. Steffen J. G. Drewe P. Hildebrand K. L.. (2011). Multiple reference genomes and transcriptomes for *Arabidopsis thaliana*. Nature 477, 419–423. 10.1038/nature10414 21874022 PMC4856438

[B15] Griffiths J. S. Tsai A. Y.-L. Xue H. Voiniciuc C. Sola K. Seifert G. J.. (2014). SALT-OVERLY SENSITIVE5 mediates Arabidopsis seed coat mucilage adherence and organization through pectins. Plant Physiol. 165, 991–1004. 10.1104/pp.114.239400 24808103 PMC4081351

[B16] Gutierrez L. Mauriat M. Guénin S. Pelloux J. Lefebvre J.-F. Louvet R.. (2008). The lack of a systematic validation of reference genes: a serious pitfall undervalued in reverse transcription-polymerase chain reaction (RT-PCR) analysis in plants. Plant Biotechnol. J. 6, 609–618. 10.1111/j.1467-7652.2008.00346.x 18433420

[B17] Hall B. G. (2013). Building phylogenetic trees from molecular data with MEGA. Mol. Biol. Evol. 30, 1229–1235. 10.1093/molbev/mst012 23486614

[B18] Hanke D. E. Northcote D. H. (1975). Molecular visualization of pectin and DNA by ruthenium red. Biopolymers 14, 1–17. 10.1002/bip.1975.360140102 51653

[B19] Harpaz-Saad S. McFarlane H. E. Xu S. Divi U. K. Forward B. Western T. L.. (2011). Cellulose synthesis via the FEI2 RLK/SOS5 pathway and cellulose synthase 5 is required for the structure of seed coat mucilage in Arabidopsis. Plant J. 68, 941–953. 10.1111/j.1365-313X.2011.04760.x 21883548

[B20] Haughn G. W. Western T. L. (2012). Arabidopsis seed coat mucilage is a specialized cell wall that can be used as a model for genetic analysis of plant cell wall structure and function. Front. Plant Sci. 3:64. 10.3389/fpls.2012.00064 22645594 PMC3355795

[B21] Horton M. W. Hancock A. M. Huang Y. S. Toomajian C. Atwell S. Auton A.. (2012). Genome-wide patterns of genetic variation in worldwide *Arabidopsis thaliana* accessions from the RegMap panel. Nat. Genet. 44, 212–216. 10.1038/ng.1042 22231484 PMC3267885

[B22] Hu R. Li J. Wang X. Zhao X. Yang X. Tang Q.. (2016). Xylan synthesized by Irregular Xylem 14 (IRX14) maintains the structure of seed coat mucilage in Arabidopsis. J. Exp. Bot. 67, 1243–1257. 10.1093/jxb/erv510 26834178 PMC4762376

[B23] Huang J. DeBowles D. Esfandiari E. Dean G. H. Carpita N. C. Haughn G. W. (2011). The Arabidopsis transcription factor LUH/MUM1 is required for extrusion of seed coat mucilage. Plant Physiol. 156, 491–502. 10.1104/pp.111.172023 21518777 PMC3177253

[B24] Kalamaki M. S. Alexandrou D. Lazari D. Merkouropoulos G. Fotopoulos V. Pateraki I.. (2009). Over-expression of a tomato N-acetyl-L-glutamate synthase gene (SlNAGS1) in *Arabidopsis thaliana* results in high ornithine levels and increased tolerance in salt and drought stresses. J. Exp. Bot. 60, 1859–1871. 10.1093/jxb/erp072 19357433 PMC2671631

[B25] Kim S. Plagnol V. Hu T. T. Toomajian C. Clark R. M. Ossowski S.. (2007). Recombination and linkage disequilibrium in *Arabidopsis thaliana*. Nat. Genet. 39, 1151–1155. 10.1038/ng2115 17676040

[B26] Kong Y. Zhou G. Abdeen A. A. Schafhauser J. Richardson B. Atmodjo M. A.. (2013). GALACTURONOSYLTRANSFERASE-LIKE5 is involved in the production of Arabidopsis seed coat mucilage. Plant Physiol. 163, 1203–1217. 10.1104/pp.113.227041 24092888 PMC3813644

[B27] Korte A. Farlow A. (2013). The advantages and limitations of trait analysis with GWAS: a review. Plant Methods 9:29. 10.1186/1746-4811-9-29 23876160 PMC3750305

[B28] Krishnakumar V. Hanlon M. R. Contrino S. Ferlanti E. S. Karamycheva S. Kim M. A.. (2015). Araport: the Arabidopsis information portal. Nucleic Acids Res. 43, D1003–D1009. 10.1093/nar/gku1200 25414324 PMC4383980

[B29] Lippert C. Listgarten J. Liu Y. Kadie C. M. Davidson R. I. Heckerman D. (2011). FaST linear mixed models for genome-wide association studies. Nat. Methods 8, 833–837. 10.1038/nmeth.1681 21892150

[B30] Loqué D. Scheller H. V. Pauly M. (2015). Engineering of plant cell walls for enhanced biofuel production. Curr. Opin. Plant Biol. 25, 151–161. 10.1016/j.pbi.2015.05.018 26051036

[B31] Macquet A. Ralet M.-C. Loudet O. Kronenberger J. Mouille G. Marion-Poll A.. (2007). A naturally occurring mutation in an Arabidopsis accession affects a beta-D-galactosidase that increases the hydrophilic potential of rhamnogalacturonan I in seed mucilage. Plant Cell 19, 3990–4006. 10.1105/tpc.107.050179 18165330 PMC2217647

[B32] Mendu V. Griffiths J. S. Persson S. Stork J. Downie B. Voiniciuc C.. (2011). Subfunctionalization of cellulose synthases in seed coat epidermal cells mediates secondary radial wall synthesis and mucilage attachment. Plant Physiol. 157, 441–453. 10.1104/pp.111.179069 21750228 PMC3165890

[B33] Millane R. P. Hendrixson T. L. (1994). Crystal structures of mannan and glucomannans. Carbohydr. Polym. 25, 245–251. 10.1016/0144-8617(94)90050-7

[B34] North H. M. Berger A. Saez-Aguayo S. Ralet M.-C. (2014). Understanding polysaccharide production and properties using seed coat mutants: future perspectives for the exploitation of natural variants. Ann. Bot. 114, 1251–1263. 10.1093/aob/mcu011 24607722 PMC4195541

[B35] Pfaffl M. W. (2001). A new mathematical model for relative quantification in real-time RT-PCR. Nucleic Acids Res. 29:e45. 10.1093/nar/29.9.e45 11328886 PMC55695

[B36] Ralet M.-C. Crépeau M.-J. Vigouroux J. Tran J. Berger A. Sallé C.. (2016). Xylans provide the structural driving force for mucilage adhesion to the Arabidopsis seed coat. Plant Physiol. 171, 165–178. 10.1104/pp.16.00211 26979331 PMC4854713

[B37] Saez-Aguayo S. Ralet M.-C. Berger A. Botran L. Ropartz D. Marion-Poll A.. (2013). PECTIN METHYLESTERASE INHIBITOR6 promotes Arabidopsis mucilage release by limiting methylesterification of homogalacturonan in seed coat epidermal cells. Plant Cell 25, 308–323. 10.1105/tpc.112.106575 23362209 PMC3584544

[B38] Saez-Aguayo S. Rondeau-Mouro C. Macquet A. Kronholm I. Ralet M.-C. Berger A.. (2014). Local evolution of seed flotation in Arabidopsis. PLoS Genet. 10:e1004221. 10.1371/journal.pgen.1004221 24625826 PMC3953066

[B39] Scheller H. V. Ulvskov P. (2010). Hemicelluloses. Annu. Rev. Plant Biol. 61, 263–289. 10.1146/annurev-arplant-042809-112315 20192742

[B40] Schindelin J. Arganda-Carreras I. Frise E. Kaynig V. Longair M. Pietzsch T.. (2012). Fiji: an open-source platform for biological-image analysis. Nat. Methods 9, 676–682. 10.1038/nmeth.2019 22743772 PMC3855844

[B41] Schwacke R. Schneider A. van der Graaff E. Fischer K. Catoni E. Desimone M.. (2003). ARAMEMNON, a novel database for Arabidopsis integral membrane proteins. Plant Physiol. 131, 16–26. 10.1104/pp.011577 12529511 PMC166783

[B42] Seren Ü. Vilhjálmsson B. J. Horton M. W. Meng D. Forai P. Huang Y. S.. (2012). GWAPP: a web application for genome-wide association mapping in Arabidopsis. Plant Cell 24, 4793–4805. 10.1105/tpc.112.108068 23277364 PMC3556958

[B43] Sullivan S. Ralet M.-C. Berger A. Diatloff E. Bischoff V. Gonneau M.. (2011). CESA5 is required for the synthesis of cellulose with a role in structuring the adherent mucilage of Arabidopsis seeds. Plant Physiol. 156, 1725–1739. 10.1104/pp.111.179077 21705653 PMC3149949

[B44] Tamura K. (1992). Estimation of the number of nucleotide substitutions when there are strong transition-transversion and G+C-content biases. Mol. Biol. Evol. 9, 678–687. 1630306 10.1093/oxfordjournals.molbev.a040752

[B45] Tamura K. Stecher G. Peterson D. Filipski A. Kumar S. (2013). MEGA6: molecular evolutionary genetics analysis version 6.0. Mol. Biol. Evol. 30, 2725–2729. 10.1093/molbev/mst197 24132122 PMC3840312

[B46] Voiniciuc C. Dean G. H. Griffiths J. S. Kirchsteiger K. Hwang Y. T. Gillett A.. (2013). FLYING SAUCER1 is a transmembrane RING E3 ubiquitin ligase that regulates the degree of pectin methylesterification in Arabidopsis seed mucilage. Plant Cell 25, 944–959. 10.1105/tpc.112.107888 23482858 PMC3634698

[B47] Voiniciuc C. Günl M. (2016). Analysis of Monosaccharides in total mucilage extractable from Arabidopsis SEEDS. Bio Protocol 6:e1801 Available online at: http://www.bio-protocol.org/e1801

[B48] Voiniciuc C. Günl M. Schmidt M. H.-W. Usadel B. (2015a). Highly branched xylan made by IRREGULAR XYLEM14 and MUCILAGE-RELATED21 links mucilage to Arabidopsis seeds. Plant Physiol. 169, 2481–2495. 10.1104/pp.15.01441 26482889 PMC4677919

[B49] Voiniciuc C. Schmidt M. H.-W. Berger A. Yang B. Ebert B. Scheller H. V.. (2015b). MUCILAGE-RELATED10 Produces galactoglucomannan that maintains pectin and cellulose architecture in Arabidopsis seed mucilage. Plant Physiol. 169, 403–420. 10.1104/pp.15.00851 26220953 PMC4577422

[B50] Voiniciuc C. Yang B. Schmidt M. Günl M. Usadel B. (2015c). Starting to Gel: how Arabidopsis seed coat epidermal cells produce specialized secondary cell walls. Int. J. Mol. Sci. 16, 3452–3473. 10.3390/ijms16023452 25658798 PMC4346907

[B51] Wang Y. Mortimer J. C. Davis J. Dupree P. Keegstra K. (2012). Identification of an additional protein involved in mannan biosynthesis. Plant J. 105–117. 10.1111/tpj.12019 22966747 PMC3558879

[B52] Weigel D. Glazebrook J. (2006). In planta transformation of Arabidopsis. CSH Protoc. 2006:pdb.prot4668. 10.1101/pdb.prot4668 22484684

[B53] Weigel D. Mott R. (2009). The 1001 genomes project for *Arabidopsis thaliana*. Genome Biol. 10:107. 10.1186/gb-2009-10-5-107 19519932 PMC2718507

[B54] Western T. Burn J. Tan W. Skinner D. J. Martin-McCaffrey L. Moffatt B. A.. (2001). Isolation and characterization of mutants defective in seed coat mucilage secretory cell development in Arabidopsis. Plant Physiol. 127, 998–1011. 10.1104/pp.010410 11706181 PMC129270

[B55] Western T. L. Young D. S. Dean G. H. Tan W. L. Samuels L. Haughn G. W. (2004). MUCILAGE-MODIFIED4 encodes a putative pectin biosynthetic enzyme developmentally regulated by APETALA2, TRANSPARENT TESTA GLABRA1, and GLABRA2 in the Arabidopsis seed coat. Plant Physiol. 134, 296–306. 10.1104/pp.103.035519 14701918 PMC316309

[B56] Winter D. Vinegar B. Nahal H. Ammar R. Wilson G. V. Provart N. J. (2007). An “electronic fluorescent pictograph” Browser for exploring and analyzing large-scale biological data sets. PLoS ONE 2:e718. 10.1371/journal.pone.0000718 17684564 PMC1934936

[B57] Yu L. Shi D. Li J. Kong Y. Yu Y. Chai G.. (2014). CSLA2, a Glucomannan synthase, is involved in maintaining adherent mucilage structure in Arabidopsis seed. Plant Physiol. 164, 1842–1856. 10.1104/pp.114.236596 24569843 PMC3982747

