# Supplementary material for: Extensive Natural Variation in Arabidopsis Seed Mucilage Structure
Source: Front Plant Sci. 2016 Jun 7;7:803. doi: 10.3389/fpls.2016.00803 (PMC4894908; doi:10.3389/fpls.2016.00803)
Supplement: Supplementary file 2 [file DataSheet1.DOCX]

**Reviewer 1**

**Independent review report submitted:**23 Mar 2016

**Interactive review activated:**11 Apr 2016

**MAIN MESSAGE**

**Q 5**

**What are the main findings reported in this manuscript?**

**Reviewer 1** | 23 Mar 2016 | 07:52

**#1**

This manuscript addresses natural variation in mucilage release in a large number of Arabidopsis accessions. Ruthenium red staining was used to compare the degree of mucilage release from the seed coat, and multiple accessions were shown to release a smaller zone of mucilage compared to the reference Col-0 accession. Compositional analysis of 25 accessions by monosaccharide profiling indicated that the levels of several sugars were changed, suggestive of a decrease in the abundance of heteromannan. GWAS analysis, using the degree of ruthenium red staining as a phenotype, identified many QTL that influence mucilage release. One of these was located close to the genomic location of the MUCILAGE-RELATED10, a gene that has previously been shown to influence galactoglucomannan synthesis. Although no specific polymorphisms were identified that might explain altered function of MUCI10 in these accessions, MUCI10 transcript was reduced and expression of Col MUCI10 was sufficient to induce the formation of Col-like mucilage. This suggests that transcriptional regulation of MUCI10 might contribute to variation in mucilage composition between different Arabidopsis accessions.

Add comment

**Q 24**

**Other comments on the materials and methods.**

**Reviewer 1** | 23 Mar 2016 | 07:52

**#1**

Line 100, Page 3: please describe clearly how many seeds were analysed for all phenotypic measurements

Each of the methods have been significantly expanded as suggested by the reviewer. Below we cite only the key responses (see manuscript for full revisions):

LINE 100, PAGE 3

Using 24-well plates, 20 to 30 seeds were mixed with 500 µL of water for 5 min. After removing the water, mucilage was stained with 300 µL of 0.01% (w/v) RR (VWR International, A3488.0001) for 5 min. The dye solution was then replaced with 300 µL of water, and an image of each well was captured with a Leica MZ12 stereomicroscope equipped with a Leica DFC 295 camera. […] a total of more than 1500 seeds were quantified for Figure 1 and Supplemental Table 1. Three biological replicates were analyzed per genotype, except only one for the HR-5 accession.

Line 132, Page 4 : How many seeds were used for mucilage extraction?

LINES 132, PAGE 4

As previously described (Voiniciuc et al., 2015b), total mucilage was extracted by vigorously mixing 5 mg of seeds with 1 mL of water (containing 30 µg of ribose as internal standard) using a ball mill, operated for 30 min at 30 Hz. After the seeds settled at the bottom of each tube, 800 µL of each supernatant was transferred to a screw-cap tube, and dried under pressurized air at 45°C. Matrix polysaccharides were hydrolyzed using 300 µL of 2 M trifluoroacetic acid for 60 min at 120°C. After a final drying step, the monosaccharides were eluted in 600 µL of water, and quantified by high-performance anion-exchange chromatography with pulsed amperometric detection (HPAEC-PAD). A serial dilution of a nine-sugar mixture (Fucose, Fuc; Rhamnose, Rha; Arabinose, Ara; Galactose, Gal; Glucose, Glc; Xylose, Xyl; Mannose, Man; Galacturonic Acid, GalA; Glucuronic Acid, GlcA; all obtained from Sigma-Aldrich) was prepared alongside the unknown samples.

Line 133, Page 4 : The use of whole mucilage samples rather than the alcohol insoluble residue (AIR) makes it difficult to assign sugars to polysaccharides rather than low molecular weight monosaccharides and oligosaccharides. 
This is will need to be clearly empahasised in the results and discussion, since there is no additional evidence provided (such as linkage analysis or immunolabelling) to indicate that the differences observed in monosaccharide composition are actually due to changes in polysaccharide abundance.

LINES 133, PAGE 4

A wide variety of extraction protocols have been used to analyze the composition of Arabidopsis seed mucilage (discussed in two reviews; North et al., 2014; Voiniciuc et al., 2015b). Total mucilage extraction (without ethanol precipitation) has been recently established as fast yet robust method to reveal changes even in low-abundance hemicellulosic polysaccharides (Voiniciuc et al., 2015a; Voiniciuc et al., 2015b). Rha and GalA (the backbone of RG I) typically represented around 90% of the total mucilage extracts (Supplemental Table 2), similar to other procedures that employed time-consuming dialysis or ethanol precipitation steps (Voiniciuc et al., 2015c).

Only seven of 25 articles surveyed (Voiniciuc et al., 2015c) precipitated mucilage polymers prior to the analysis of monosaccharide composition, AIR preparation of initial mucilage extracts from Ws (Arsovski et al., 2009) and Col-0 (Kunieda et al., 2013) accessions resembled the composition of our total mucilage extracts.

Line 140, Page 3: GWAS analysis is a powerful tool to identify genomic regions that influence a specific trait across a population of related individuals. Please describe how the significance threshold was determined in Figure 3. Also, limitations in the LMM methodology in correcting for population structure mean that several methods should be considered (eg. Improving the Power of GWAS and Avoiding Confounding from Population Stratification with PC-Select, George Tucker, Alkes L. Price, Bonnie Berger, Genetics, 2014 vol. 197 no. 3 1045-1049) when assessing QTL data. Does the GWAPP or LMM analysis undertaken in this study take population structure?

LINE 140, PAGE 3. The dashed line in Figure 3A represents the 5% false discovery rate threshold obtained using the Benjamini–Hochberg–Yekutieli multiple testing procedure (Benjamini and Yekutieli, 2001), assuming arbitrary dependence between SNPs.

Indeed population structure and other confounding effects are a major concern, this is why we first used the standalone tool FaST-LMM as it has been described as taking these into account very efficiently, we changed the wording as follows:

Since linear mixed models (LMM) are becoming the method of choice to correct for population structure and relatedness (Eu-ahsunthornwattana et al., 2014), we first performed GWAS analysis using FaST-LMM (Supplemental Figure 3). As we obtained qualitatively similar results in the GWAPP web application ([http://gwapp.gmi.oeaw.ac.at](http://gwapp.gmi.oeaw.ac.at/); Seren et al., 2012), which can be conveniently accessed by other users, we then used in this tool for further data visualization.

Line 196: Using "non-expressing" transgenics rather than null-segregants does not appear to be a common method when comparing the effects of a transgene. Are you sure that a lack of leaf expression always correlates with a lack of expression in seeds?

***Line 196:*** We previously demonstrated that the 35S-driven expression of MUCI10 tagged with yellow super fluorescent protein (35S:MUCI10-sYFP) could at least partially rescue the muci10-1 T-DNA mutant defects, unlike the 35S:sYFP control (Voiniciuc et al., 2015b). In all the transformed lines we examined, the detection of fluorescent punctae in rosette leaves correlated with the expression of MUCI10-sYFP in other tissues where 35S promoter is active, such as the seed coat. Conversely, in our experience, some plant survived the Basta selection but did not display any MUCI10-sYFP fluorescent signals in leaves or other tissues examined (eg. seed coat).

**Q 27**

**Have the author(s) included all the information required to reach the conclusions?**

**Reviewer 1** | 23 Mar 2016 | 07:52

**#1**

No
Line 211: Presumably the authors collected seed area measurements for the 280 accessions that were used in GWAS analysis. Although there appeared to be no correlation between seed area and mucilage release in a subset of lines, the authors might consider using total seed area as an additional trait for GWAS to confirm that there is no overlap between QTL influencing seed area and ruthenium red staining.

LINE 211

Seed area measurements are not available for the complete set, since the initial screen of around 280 accessions was only focused on identifying accessions in semi-quantitative changes in RR-stained mucilage capsule size. Although mucilage and did not correlate in the subset of accessions selected in Table 1, additional accessions showed larger seeds and mucilage capsules compared to Col-0 (1.2 score in Supplemental Table 3). We now include the complete mucilage phenotypic data for all accessions examined in Supplemental Tables 3A and 3B.

Line 212: Are there differences in seed coat (i.e. epidermal) morphology between the accessions that might also explain the differences in mucilage release?

LINE 212

Scanning electron microscopy was previously used to examine the seed coat epidermal cell morphology of three of the mucilage-modified accessions, including Lc-0 (which had the most severe reductions in Rha and GalA). ***The Lm-2, Ri-0 and Lc-0 accessions showed no obvious defects in seed coat morphology (Voiniciuc et al., 2015b).***

Line 231: It is not clear to me whether the Gal and Man differences are due solely to variations in polysaccharide abundance because total mucilage extract was used in the chemical analysis. The authors should consider testing the the alcohol insoluble fraction to confirm differences in polysaccharide abundnace. Also, since there is an antibody available for GGM, the authors should also consider testing the distribution/abundance of GGM in the seed coat mucilage of the accessions to determine whether the differences are due to changes in overall abundance or changes in GGM sub-domains within the mucilage.

LINE 231

We significantly revised the results and discussion to clarify this point. Lm-2, Ri-0 and Lc-0 accessions were recently shown to be heteromannan-deficient, unlike the Col-0 reference, based on immunolabeling of mucilage capsules with the LM21 monoclonal antibody (Voiniciuc et al., 2015b). These three variants phenocopied the mucilage LM21 labeling of the csla2 and muci10 mutants with impaired GGM synthesis.

Line 265: A lack of cell wall domain does not mean the genes are not involved in seed coat development. I suggest examining the expression patterns of the genes in this region using public databases to at least determine if they are seed expressed or even seed-coat expressed. Although MUCI10 is a very promising candidate, other linked genes may be involved.

LINE 265

This section has now been expanded to include additional GWAS candidate genes:

“close examination of the highest peak on Chr2 (arrow in Figure 3A) revealed an association with *MUCI10* (Figure 3B), which directly affects GGM synthesis and mucilage structure (Voiniciuc et al., 2015b). In contrast to *MUCI10*, no *CSLA* or *MANNAN-SYNTHESIS RELATED* genes (Wang et al., 2012) were found within 1 million bases (representing 100 times the average linkage disequilibrium decay in Arabidopsis; Kim et al., 2007) of the GWAS peaks in Table 2. Although *MUCI10* is the only cell wall-related gene near the Chr2 peak, two other genes are expressed in seeds according to the public microarray data (Winter et al., 2007; Belmonte et al., 2013). While At2g22870 (*EMBRYO DEFECTIVE 2001*, *EMB2001*) is primarily expressed in the embryo, At2g22910 (*NAGS1*), which is predicted to facilitate amino acid synthesis (Kalamaki et al., 2009) is expressed at low levels throughout seed coat development (Supplemental Figure 4). Since *MUCI10* was the only gene predicted by GWAS known to affect the synthesis of Man-containing polymers, and most of the mucilage-modified natural variants phenocopied the *muci10-1* mutant defects (Figure 1; Figure 2), we focused on this very promising candidate for further experiments.”

Line 289: MUCI10 and CslA transcript were tested for changes in gene expression in several lines. An important control will be to test the abundance of a other cell wall genes ie. CesA, PMEI.... to see if it is a specific down-regulation of these GGM genes or is a general change in cell wall polyscaccharide biosynthesis.

LINE 289

We followed the reviewer’s recommendation:

***“***To check if these accessions specifically affect GGM-related genes or hemicellulose biosynthesis in general, we also analyzed the expression of *IRX14*, which is the critical for the elongation of xylan polymers in seed mucilage (Voiniciuc et al., 2015a). While Col-0, Ema-1 and Sei-0 siliques had similar *IRX14* transcript levels, Le-0 and Mz-0 showed higher xylan gene expression (Figure 5B). This suggests that Sei-0 has a specific down-regulation of GGM-related genes, but Le-0 and Mz-0 have broader transcriptional changes that also affect xylan synthesis. “

Line 294: The mucilage phenotypes might be stable within inbred accessions, but were they crossed to Col-0 to see if the phenotypes could actually be found segregating in progeny? This is a more appropriate test to determine heritability.

LINE 294

As the reviewer suggested, future studies should include crosses between the accessions with modified mucilage properties and the Col-0 reference to determine the inheritance patterns of the observed traits. We now emphasize this in the discussion:

“Since the accessions identified in this study fall into five phenotypic groups that are still loosely defined (Figure 1), future research should examine their cell wall defects using additional techniques such as linkage analysis and immunolabeling to elucidate their effects on polysaccharide structure. In addition, backcrosses to Col-0 will be necessary to establish the segregation pattern of each trait.

**Other comments on the results.**

**Reviewer 1** | 23 Mar 2016 | 07:52

**#1**

Line 230: the GGM acronym is not explained

LINE 230

Galactoglucomannan (GGM) is now defined in line 60, and abbreviated afterwards.

Supplementary figure: I suggest adding a supplementary table indicating regions containing all putative QTL (7 of them?), the linked markers and lists of candidate genes within a 5cM region flanking the QTL.

We add a new table in the main text (Table 2) that outlines the precise positions of all the SNPs above the 5% FDR threshold, as well as the identity of the nearest genes. Users can easily find additional GWAS candidates by browsing the interactive GWAPP results (using the input data in Supplemental Table 3A), or by searching TAIR (Restrict by Map Locations; <https://www.arabidopsis.org/servlets/Search?type=gene&action=new_search>).

**Q 31**

**Are the conclusions justified?**

**Reviewer 1** | 23 Mar 2016 | 07:52

**#1**

No
Line 337: Without linkage analysis and/or immunocytology, it is not correct to claim that heteromannan polysaccharide is changed in abundance. Monosaccharide analysis on whole mucilage samples reveals details of free sugars as well as high molecular weight sugars. Unless AIR was used and it has not been described, please address this throughout the manuscript.

***LINE 337***

***We significantly revised the results and discussion to comply with the reviewer’s comment. The major changes are highlighted in yellow in the text.***

Lines 373-382: The QTL near MUCI10 is clear, indicating that that genetic polymorphisms in this region contribute to the variations in mucilage release. If altered transcriptional regulation of MUCI10 is the cause of the phenotypes, then this can only be explained by missing binding sites in the promoter of MUCI10 or altered activity of TFs underlying other QTL. Putative TF binding sites shoiuld be analysed in the MUCI10 promoter using PlantPAN (http://plantpan2.itps.ncku.edu.tw/) and then compared between sequenced accessions to determine if anything is missing. Also, are there any TFs underlying other QTL? A list of candidates would be helpful (see above)

LINES 373-382

We performed the analysis recommended by the reviewer: “Based on the biochemical profiling (Figure 1) and the GWAS results (Figure 3), we hypothesized that altered expression of *MUCI10* may contribute to natural variation in mucilage structure. The Plant Promoter Analysis Navigator (PlantPAN; <http://PlantPAN2.itps.ncku.edu.tw>; Chow et al., 2016) was used to identify transcription factor binding sites upstream of *MUCI10*. We filtered a large list of putative regulators of *MUCI10* based on their proximity to the high GWAS peaks (Figure 3A, Table 2). Only nine of transcription factors had conserved motifs upstream of *MUCI10* that were affected by the large sequencing gaps or other genetic polymorphisms in at least one of the accessions (Supplemental Table 4). One of these candidates (At2g22800), which encodes the homeobox protein HAT9, was in nearby *MUCI10* (38075 bp away) and was also up-regulated in the seed coat at the developmental stage of secondary cell wall production (Supplemental Figure 4). “

_____________

**Reviewer 2**

**Independent review report submitted:**11 Apr 2016

**Interactive review activated:**11 Apr 2016

You can post and reply to comments within this review forum here. On completion, ensure that you click on **Submit all comments** in order to alert the other participants to your changes.

**S6. OTHER COMMENTS**

**Q 40**

**Please add here any further comments on this manuscript.**

**Reviewer 2** | 11 Apr 2016 | 18:37

**#1**

Review of Voiniciuc et al. manuscript submitted to Frontiers in Plant Science

Extensive Natural Variation in Arabidopsis Seed Mucilage Structure

Summary:

This manuscript describes analysis of seed mucilage structure/content of a large collection of natural accessions of Arabidopsis thaliana. The accessions were initially compared to the Col-0 accession in terms of ruthenium red (RR) staining of pectic mucilage components, and this screen resulted in the identification of about 50 accessions with RR-stained mucilage capsules differing from Col-0. Twenty-five of these accessions, with smaller than WT RR-stained capsules were subjected to detailed characterization (pontamine fast scarlet staining, birefringence, monosaccharide analysis). Characteristics of these 25 accessions are described in this manuscript. Some of the main findings are summarized below:

• All 25 accessions with reductions in RR staining area also exhibited decreased S4B staining and birefringence. There was no clear correlation between the degree of reduction of RR-staining vs S4B and/or birefringence. 
• Monosaccharide content of total seed mucilage varied considerably among the 25 accessions with reduced RR staining area. Variation of monosaccharides present in galactoglucomannan was particularly evident, with galactose and mannose content of accessions ranging from significantly less to significantly more than the Col-0 control. Thus, while all tested accessions presented reduced RR-staining, substantial variation of the underlying carbohydrate content of the seed mucilage capsule was observed. 
• A genome-wide association study linked a region of chromosome 2 containing the MUCI10 gene to RR-staining defects. This gene encodes a GT34 galactosyltransferase that has previously been implicated galactosylation of seed mucilage GGM.
• Analysis of the MUCI10 gene in the accessions with decreased RR staining resulted in the identification of a single non-synonymous SNP (R109H). It isn’t clear in which accession(s) this SNP is present.
• Additional SNPs and variations were identified in the intergenic region upstream of MUCI10 also were identified in preliminary genome sequence data. The significance of these intergenic variations was not clear.
• MUCI10 and CSLA2 transcript abundance was characterized in three accessions with decreased GGM content (similar to muc10-1 insertion mutant). These lines displayed wild-type levels of CSLA2 transcript, but significantly decreased levels of MUCI10 transcript.
• MUCI10 and CSLA2 transcript abundance also was characterized in three accessions with increased mannose content. One of these accessions (Sei-0) displayed decreased levels of both transcripts, while the other two (Le-0 and Mz-0) displayed significantly increased levels of both transcripts. 
• Complementation of the RR-staining and GGM sugar content deficiencies of the Ema-1 accession was achieved using a 35S:MUCI10-sYFP construct. This construct did not complement these deficiencies in several other accessions. 

Specific questions/comments/suggestions about the manuscript:

1) Proposed wording change (pg. 2, line 53): …of the total mucilage produced, called adherent mucilage, remains attached…

POINT 1

We rephrased this: “Only 35% of the total RG I produced is part of the adherent mucilage layer that remains attached to seeds after gentle shaking in water (Voiniciuc et al., 2015c).”

2) Comment (pg. 6, line 235-236): glucose also is a building block of GGM

POINT 2

We rephrased this as suggested: “Previous mucilage immunolabeling experiments indicate that Lm-2, Ri-0, and Lc-0 are deficient in GGM polymers (Voiniciuc et al., 2015b), which consist of Gal, Glc, and Man subunits. […]While altered GGM abundance should result in proportional changes in Glc and Man, 16 mucilage-modified accessions had inconsistent spikes in Glc (coefficient of variation above 0.4), which were not observed for other mucilage components (Supplemental Table 2). Glc represented only 1% of Col-0 mucilage extracts, but was at least 5 times more abundant in 23 of the 137 mucilage samples analyzed for Supplemental Table 2. These dramatic increases in Glc did not strongly correlate with the genotype and were not detected in other growth batches, while the changes in mucilage Gal and/or Man levels were stable for most accessions (data not shown). “

3) Proposed wording change (pg. 7, line 260): …we used this web application… (delete “in”)

POINT 3: Fixed.

4) Question/comment (pg. 7, line 269): in which accession(s) was the R109H SNP present? Was there a relationship between this particular SNP and mucilage phenotype?

POINT 4

We expanded this results section as follows:

“Sei-0 had a P119H substitution (Proline at position 119 changed to Histidine), while nine other accessions (including the Man-rich Le-0 variant) contained a non-synonymous SNP that induced R109H (marked with a red H in Figure 3C). Although both changes result in amino acids with distinct chemical properties (Betts and Russell, 2007), these SNPs occur between the MUCI10 transmembrane and galactosyltransferase domains annotated in the ARAMMEMNON database (http://aramemnon.botanik.uni-koeln.de; Schwacke et al., 2003). While the SNPs in the MUCI10 coding sequence do not have obvious deleterious effects, the preliminary 1001 Genome data suggests that many of the mucilage-modified accessions have large gaps (see pink bars in Figure 3C) and other polymorphisms in the large intergenic region upstream of the MUCI10 start codon. We examined if the polymorphisms in MUCI10 correlate with the mucilage chemotypes reported in Figure 1, but did not identify sets of mutations that were consistent with the phenotypes. Indeed, the Man-rich accessions (Dr-0, Le-0, Mz-0, Sei-0), which were collected from distinct parts of Europe (Figure 4A), clustered with Man-deficient accessions in a phylogenetic tree of MUCI10 coding and upstream sequences (Figure 4B).”

5) Question/comment (pg. 7, lines 274-275): was the MUCI10 upstream intergenic region more of a hotspot for variation compared to other random areas of the genome?

POINT 5

Large sequencing gaps in the intergenic regions are not unique to MUCI10, and have been observed in other candidate genes we examined. In addition, the MUCI10 peak region did not seem to exhibit specific nucleotide diversity peaks or troughs in study of recombination and linkage disequilibrium in Arabidopsis thaliana (Kim et al., 2007).


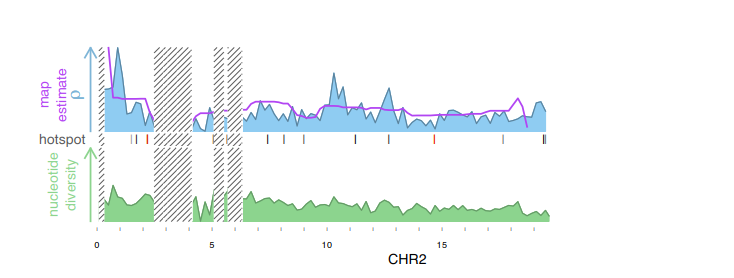


We have however significantly expanded the results section to describe other genes predicted by GWAS and why we focused on MUCI10:

***“Since MUCI10 was the only gene predicted by GWAS known to affect the synthesis of Man-containing polymers, and most of the mucilage-modified natural variants phenocopied the muci10-1 mutant defects (Figure 1; Figure 2), we focused on this very promising candidate for further experiments.”***

6) Edit (pg. 16, Figure 1A): Knox-18 should be angled, like the other accession labels on the heatmap

POINT 6

We fixed this and combined the heatmaps from Figure 1 and 2 into a simpler format.

7) Question/comment (pg. 17, Figure 2): is the Glc value (1312%) of the Pla-0 accession correct, or is this a typo?
POINT 7

This is not a typo. Some of our mucilage extracts from accessions showed surprisingly large spikes in Glc. We now describe this in more detail in the results:

“While altered GGM abundance should result in proportional changes in Glc and Man, 16 mucilage-modified accessions had inconsistent spikes in Glc (coefficient of variation above 0.4), which were not observed for other mucilage components (Supplemental Table 2). Glc represented only 1% of Col-0 mucilage extracts, but was at least 5 times more abundant in 23 of the 137 mucilage samples analyzed for Supplemental Table 2. These dramatic increases in Glc did not strongly correlate with the genotype and were not detected in other growth batches, while the changes in mucilage Gal and/or Man levels were stable for most accessions (data not shown).“

8) Question/comment (pg. 17, Figure 2): Does it make sense to compare the values to wild-type Col-0? For example, an accession like Ra-0 has 79% total mucilage content of Col-0, but 99% man content of Col-0. As displayed in this figure, the man content of Ra-0 is neither increased or decreased compared to Col-0. On the other hand, if I am correctly interpreting these data, seed mucilage of Ra-0 is enriched in man relative to seed mucilage of Col-0.

POINT 8

The reviewer correctly interpreted the relative values in Figure 2. We normalized the absolute amounts (ug sugar/mg seed) of each monosaccharide in the mucilage-modified accessions to the Col-0 values in order to present the mucilage composition of 28 genotypes in a heatmap that indicates which components are particularly affected. Ra-0 has produces Man levels similar to the Col-0, but around 20% less Rha, GalA and Xyl. To simplify the results, we now combine the heatmaps from Figure 1 and 2 into one, and present some of the monosaccharide data only in Supplemental Table 2.

9) Question/comment (pg. 18, Figure 3): The authors focus on a region of chromosome 2 containing the MUCI10 gene that was identified using a genome wide association study. There appeared to be several other chromosomal regions above the 5% false discovery threshold. Have the authors investigated whether any of these regions contain genes likely to be involved with mucilage carbohydrate biosynthesis (e.g., any of the other eight CSLA genes or other CSL genes, At4G37690 - GT6, α-galactosidase genes, MSR1/2 genes, etc.). The authors should elaborate their description of the GWAS results and corresponding discussion.

POINT 9

We now describe this: “In contrast to MUCI10, neither the CSLA genes nor the MANNAN-SYNTHESIS RELATED (MSR) genes, which are also required for heteromannan synthesis (Wang et al., 2012), were found within 1 million bases of the GWAS peaks.”

10) Question/comment (pg. 20, Figure 6): In panel E, seeds of which Ema-1 complementation line are shown? Five different transformed lines were characterized in panels A & B. Was the RR staining pattern similar in all five complementation lines, thereby making the data shown in E, representative?

POINT 10

The selected images were representative of each genotype, but we added six new panels to Figure 6 that show the RR staining pattern of untransformed Ema-1 seeds, as well as all the transformed lines.

11) Question/comment (pg. 22, Table 1): Do the elevation values represent the actual elevation of the site at which the seeds of the accession were collected? Or were these values based on an elevation value from the area. If the values do not correspond to the actual site of collection, the reviewed believes they should not be included in the table.

POINT 11

As suggested by the reviewer, we removed the elevation values, since most accessions were collected without precise GPS coordinates, and their location was estimated based on the nearest city.
