# Supplementary material for: Extensive Natural Variation in Arabidopsis Seed Mucilage Structure
Source: Front Plant Sci. 2016 Jun 7;7:803. doi: 10.3389/fpls.2016.00803 (PMC4894908; doi:10.3389/fpls.2016.00803)
Supplement: Supplementary file 3 [file Image1.PDF]

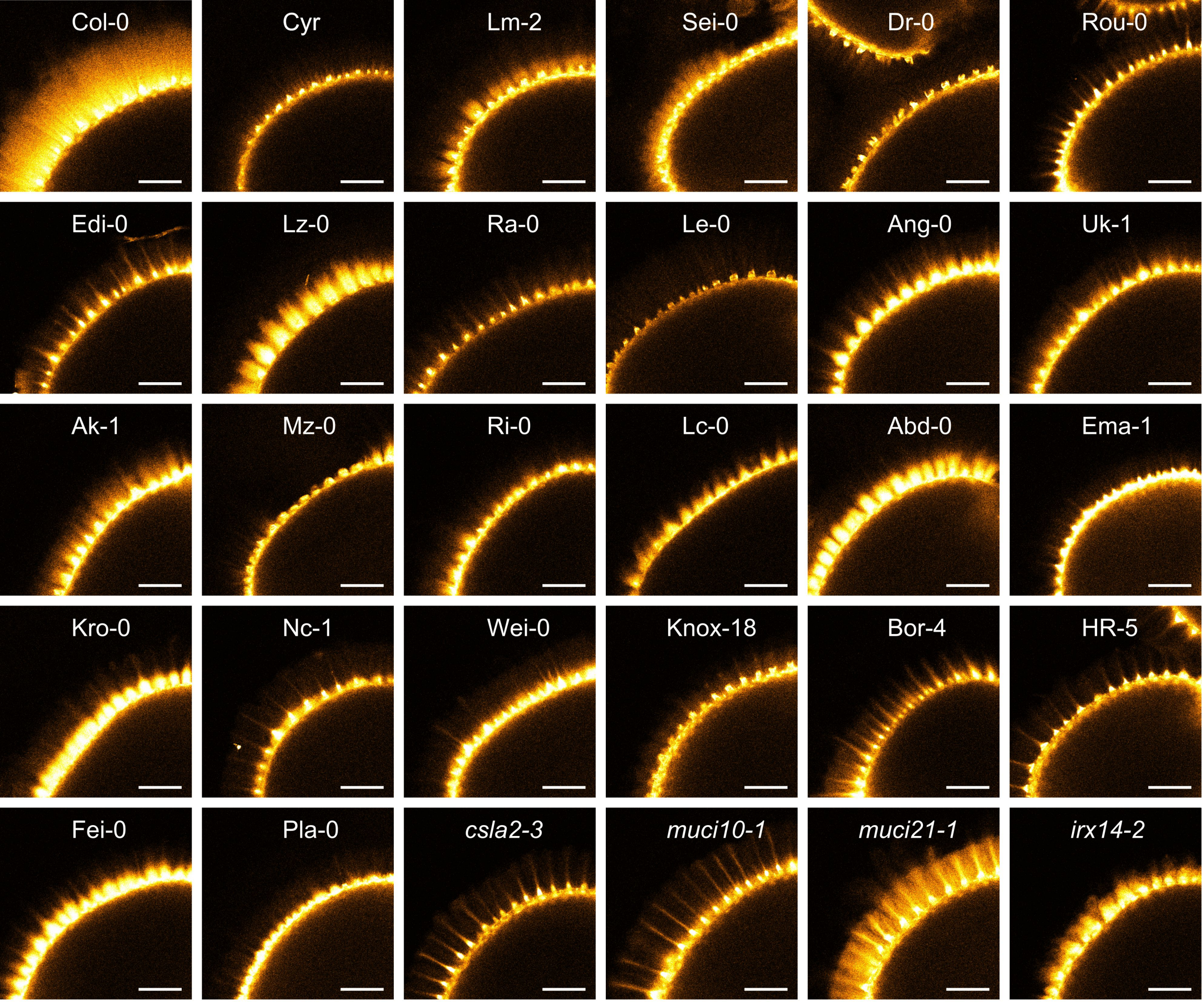

Supplemental Figure 1. Distribution of S4B-stained cellulose in mucilage. Fluorescent signals were visualized using the Orange Hot look-up table in Fiji. Mucilage-modified accessions are sorted as shown in Table 1. Scale bars = 100 µm.

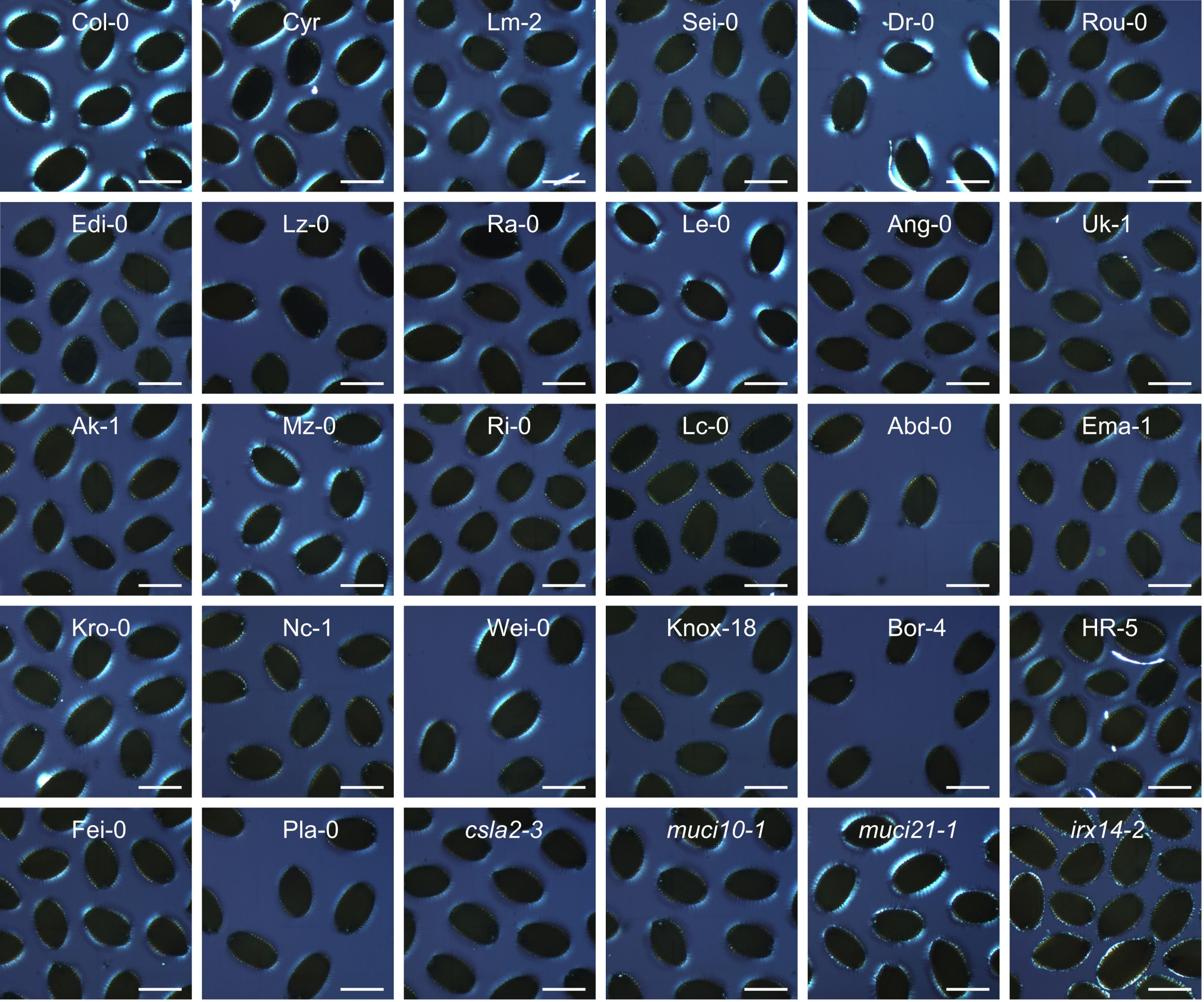

Supplemental Figure 2. Birefringence of crystalline structures in mucilage. Water-hydrated seeds for Col-0, 25 mucilage-modified accessions (same order as Table 1) and four T-DNA insertion mutants were examined with polarized light. Scale bars = 0.9 mm.

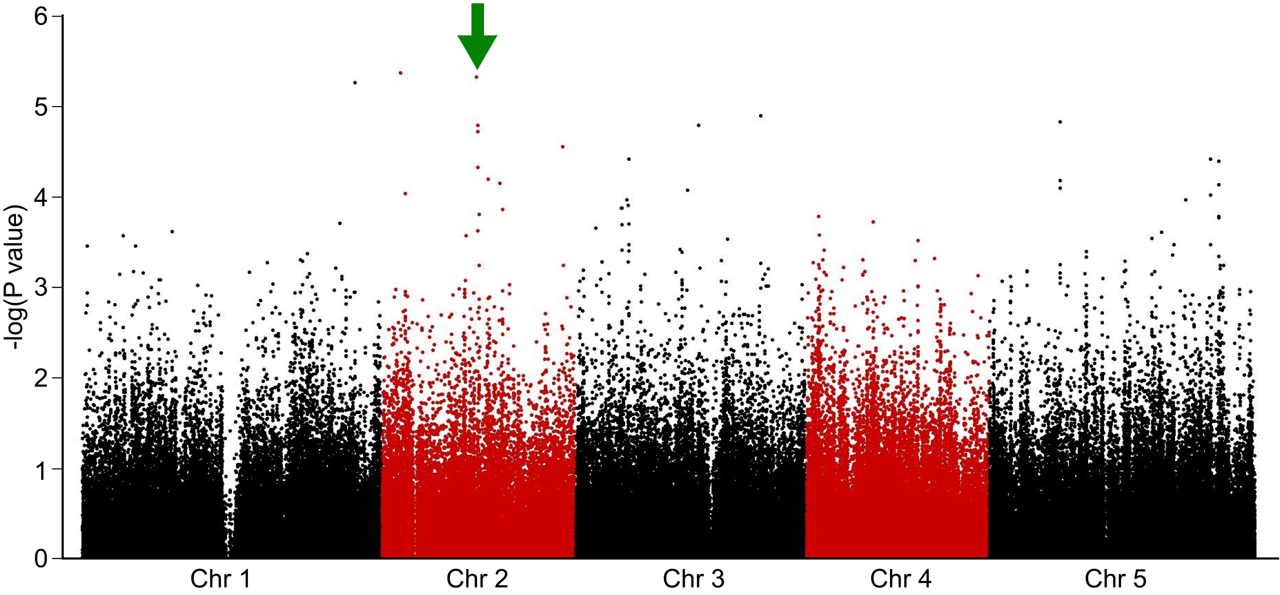

Supplemental Figure 3. Manhattan plots of GWAS analysis conducted using FaST-LMM. Associations between SNPs and semi-quantitative RR staining traits of 175 Arabidopsis accessions. Graph shows the result of the FaST-LMM analysis (version 2.07; Lippert et al., 2011), using “exact” inference and excluding SNPs with a minor allele frequency below 0.05. The green arrow indicates a peak near *MUCI10* on Chromosome 2 (Chr2), consistent with Figure 3A.

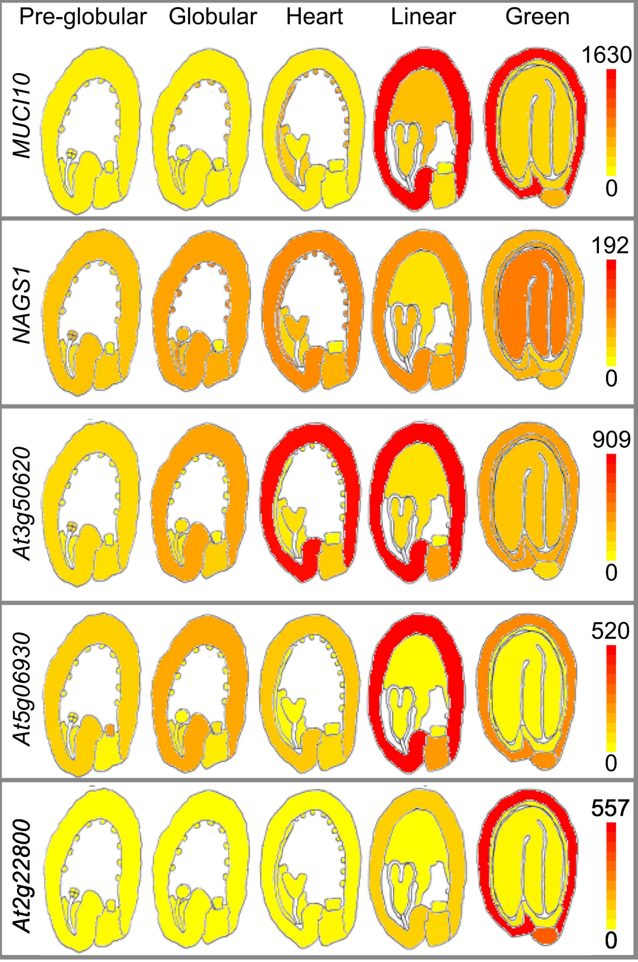

Supplemental Figure 4. Seed coat expression profiles of GWAS candidate genes. Unlike *MUCI10*, *NAGS1* is not up-regulated at the stage of secondary cell wall production. ATH1 microarray data was visualized with the eFP Browser (Winter et al., 2007; Belmonte et al., 2013).
